# Calpain 3 Is a Rapid-Action, Unidirectional Proteolytic Switch Central to Muscle Remodeling

**DOI:** 10.1371/journal.pone.0011940

**Published:** 2010-08-04

**Authors:** Antoine de Morrée, David Lutje Hulsik, Antonietta Impagliazzo, Herman H. H. B. M. van Haagen, Paula de Galan, Alexandra van Remoortere, Peter A. C. 't Hoen, GertJan B. van Ommen, Rune R. Frants, Silvère M. van der Maarel

**Affiliations:** 1 Center for Human Genetics, Leiden University Medical Center, Leiden, The Netherlands; 2 Department of Cellular Architecture and Dynamics, Institute of Biomembranes, Utrecht University, Utrecht, The Netherlands; 3 Department of Parasitology, Leiden University Medical Center, Leiden, The Netherlands; McMaster University, Canada

## Abstract

Calpain 3 (CAPN3) is a cysteine protease that when mutated causes Limb Girdle Muscular Dystrophy 2A. It is thereby the only described Calpain family member that genetically causes a disease. Due to its inherent instability little is known of its substrates or its mechanism of activity and pathogenicity. In this investigation we define a primary sequence motif underlying CAPN3 substrate cleavage. This motif can transform non-related proteins into substrates, and identifies >300 new putative CAPN3 targets. Bioinformatic analyses of these targets demonstrate a critical role in muscle cytoskeletal remodeling and identify novel CAPN3 functions. Among the new CAPN3 substrates are three E3 SUMO ligases of the Protein Inhibitor of Activated Stats (PIAS) family. CAPN3 can cleave PIAS proteins and negatively regulates PIAS3 sumoylase activity. Consequently, SUMO2 is deregulated in patient muscle tissue. Our study thus uncovers unexpected crosstalk between CAPN3 proteolysis and protein sumoylation, with strong implications for muscle remodeling.

## Introduction

Regulated proteolysis is an indispensible mechanism for cell physiology. By controlling protein degradation, protein levels can be modulated without generating toxic waste products. Proteases are classified according to their reactive site residues into clans and families. The clan of cysteine proteases includes the Calpain family. Calpains are non-denaturing proteases that respond to local changes in calcium. Amongst their many functions, Calpains are involved cytoskeleton remodeling [1].

Calpain 3 (CAPN3) is a muscle specific Calpain family member. When mutated it causes Limb-Girdle Muscular Dystrophy (LGMD) 2A (OMIM#253600, the most common form of LGMD in many populations). [2] It is currently not known how CAPN3 functions and how its dysfunction causes disease. CAPN3 shares ∼50% identity with Calpain 1 and 2 (MIM*114220 and *114230, respectively)[3] on amino acid level, but is unique in that it contains three specific insertion sequences (NS, IS1 and IS2) which have to be autolytically removed for CAPN3 to become proteolitically active. Like Calpains 1 and 2 CAPN3 has a calcium-sensing domain, which operates with nanomolar sensitivity and influences protease activation and activity [4]. In muscle tissue most, but not all, of the CAPN3 protein can be found in an inactive form bound to the sarcomere. Upon stimulation, CAPN3 both activates and deactivates itself rapidly through autolysis of the insertion sequences. After removal of the IS1 sequence the protein continues as a proteolytically active intramolecular heterodimer. Subsequent continued autolysis results in protease deactivation. *In vitro* studies have shown that within 10 minutes autolysis can result in its total destruction [3], but this depends on free [Ca2+] [4]. Due to this instability little is known of its substrates and its mechanism of action, but it has been hypothesized that CAPN3 must have a very short range activity. [5] Intriguingly, CAPN3 cleavage has been suggested to be both very specific[4] and very general[5] in nature. Defining the motif for CAPN3 mediated cleavage would therefore provide important insight in the role of this enigmatic protein in local tissue remodeling.

Previous proteomic[6] and yeast-two-hybrid studies have identified several substrates that collectively hint at a role for CAPN3 in cytoskeleton remodeling[7], [8], just like its family members. However, the number of characterized substrates is small, and does not allow for a comprehensive investigation into CAPN3 cleavage and function. Large peptide-based libraries have been used to determine protease cleavage motifs of Calpain 1 and 2. [9] However, it has been postulated that Calpain proteases not only recognize a primary sequence motif, but also a three-dimensional fold[10], obstructing cleavage motif identification by this strategy as peptide libraries likely lack essential folding properties. Multiple Calpain 1 and 2 substrates have been shown to contain a PEST domain. Deletion of this domain renders certain substrates insensitive to proteolytic cleavage. [11] However, in many other cases the PEST site has no influence on Caplain-mediated proteolysis[12], leaving the role of these sequences uncertain. Comparison of experimentally determined cleavage sites of Calpains 1 and 2 showed several amino acids to be overrepresented, but this proved inconclusive[13] and does not compare well with peptide-based results. [9] In fact, some proteases are able to cleave after every possible amino acid, [14] indicating that in addition to the primary sequence, surface exposure and peptide flexibility are important determinants of Calpain substrate recognition and cleavage [14].

From this we hypothesized that a cleavage motif might only be identified from peptide analysis, when the determinants of presentation and flexibility are taken into account.

There are many methods in literature for predicting substrates and cleavage sites by proteases. [15] In this investigation we used a bio-informatics approach to derive a CAPN3 substrate target sequence. We show that the CAPN3 consensus cleavage motif that we have identified can transform non-substrate proteins into substrates and can be used to predict and identify new substrates. Our data supports CAPN3 to be a regulator of rapid local changes in cytoskeletal protein content, identifies novel roles for CAPN3, and highlights CAPN3′s role as a molecular switch in muscle remodeling.

## Results

### Identification of a functional CAPN3 cleavage motif

We hypothesized that CAPN3 substrates harbor a uniform cleavage motif. From literature we identified several substrates (Table 1). [8], [16], [17] As substrate criterion we only considered proteins for which the actual cleavage fragments had been experimentally tested. We screened these proteins for the presence of similar sequences based on amino acid structure using the computer algorithm TEIRESIAS. [18] This yielded a motif of 10 amino acids with 4 key residues: [LIMV]_1_-X_2_-X_3_-X_4_-X_5_-[LIMV]_6_-X_7_-X_8_-[LIMV]_9_-[DE]_10_ in which X represents any amino acid (Table 1).

**Table 1:** Overview of CAPN3 substrates and tested peptides.

Described substrates					
[LIMV]X(4)[LIMV]X(2)[LIMV][DE]					
Motif	Cleaved	Prot ID	Gene ID	Location	Protein
V PSAN I EG L E	+	Q09666	*AHNAK*	356–381	AHNAK-N (16)
L PAIH V EG L D	+/−	Q09666	*AHNAK*	5124–5159	AHNAK-C1 (16)
L PGIG V QG L E	+/−	Q09666	*AHNAK*	5468–5503	AHNAK-C2 (16)
V EECY V SE L D	+	Q14315	*FLNC*	2451–2486	Filamin C (15)
I TKEC V MR V D	+	Q9Y490	*TLN1*	330–365	Talin (8)
I KKKL V QR L E	+	Q9Y490	*TLN1*	907–942	Talin (8)
L YPEP V RV L E	+	Q8WZ42	*TTN*	1843–1878	Titin_1607–2167_ (8)
V RYDG I HY L D	+	Q8WZ42	*TTN*	1887–1922	Titin_1607–2167_ (8)
L TTER L VH V D	+	Q8WZ42	*TTN*	795–830	Titin_741–948_ (8)
No motif		Q8WZ42	*TTN*	No hit	Titin_952–1540_ (8)
V KYEG I GP V D	+/−	O60504	*SORBS3*	117–152	Vinexin (8)
V WYFG L HY V D	+/−	P15311	*EZR*	30–65	Ezrin (8)
I GEET V IT V D	ND	P21333	*FLNA*	1664–1699	Filamin A (8)
L EECY V TE I D	ND	P21334	*FLNA*	2373–2408	Filamin A (8)
V PDVS L EG **P** E	−	Q09666	*AHNAK*	1293–1302	AHNAK-R (16)

Mutations in motif sequence			
AHNAK-N peptide		FLNC peptide	
Motif	Cleaved	Motif	Cleaved
V PSAN I EG L E	+	V EECY V SE L D	+
V PSAN I EG **P** E	−	V EECY V SE **P** D	−
V PSAN I EG L **A**	−	V EECY V SE L **A**	+/−
V PSAN I EG L **K**	+/−	V EECY V SE L **K**	ND
**A** PSAN I EG L E	−	**A** EECY V SE L D	−
V PSAN I EG **A** E	+	V EECY V SE **A** D	ND
V PSAN I EG M E	+	V EECY V SE **M** D	ND
V PSAN I EG **I** E	+	V EECY V SE **I** D	ND
V PSAN **A** EG L E	+	V EECY **A** SE L D	ND
V PSAN **A** EG **A** E	+	V EECY **A** SE **A** D	+/−
V PSAN **K** EG L E	−	V EECY **K** SE L D	−
V PSAN **E** EG L E	+/−	V EECY **E** SE L D	−
V ESAN I EG **E** E	ND	V EECY V SE **E** D	−
V P**.**AN I EG L E	+/−	V E**.**CY V SE L D	−
**D** PSAN **D** EG **D** **D**	−	V **P**ECY V SE L D	+

Predicted substrates			
[LIMV]X(3,4)[LIMV]X(2)[LIMV][DE]			
Motif	Cleaved	Protein ID	Gene ID
V GIQ. L PE V E	+	Q09666	*AHNAK*
L DLRS I KE V D	+	P20810	*CAST*
M PSFK M PK V D	+	Q8IVF2	*AHNAK2*
L IARM V RN M D	−	P20807	*CAPN3*
L IARM V R**G** M D	+/−	P20807	*CAPN3* mutation
L EKK. V LY V D	+	P20807	*CAPN3*
I YPAT I AS I D	+/−	Q16637	*SMN1*

[VLM][PGED][SAGEKL]{G}X(0,1)[VAIML][ESPQLK][GKEY][VLA][ED]			
Motif	Cleaved	Protein ID	Gene ID
LPAmkAKGLE	+	Q10567	*AP1B1*
LPAvkAKGLE	+	P63010	*AP2B2*
LGLvpVEEVD	+	Q9BWV1	*BOC*
MEGtvAPEAE	−	P49711	*CTCF*
LGSfnLEKVE	+	Q13426	*XRCC4*
VPLssAEEAE	+	Q8WZA1	*POMGNT1*
VPAayVKKLD	+	Q13813	*SPTAN1*
LGAlfLEYVD	+	Q5TBA9	*FRY*
LPApcASKVE	+	Q9UBL6	*CPNE7*
VDEeaMEKAE	+	Random	*Random*
VDLv.MQKVE	+	Q5VT25	*CDC42BPA*
LGEepLLEVE	+	Q9UNK9	*ANGEL1*
LPStcLQKVE	+	Q00975	*CACNA1B*
LGEvqAEKAD	−	O94911	*ABCA8*
LGGldVLEAE	+	Q5SGD2	*PPM1L*
LEEk.LKEAE	+/−	P07951	*TPM2*

**Figure 1:**
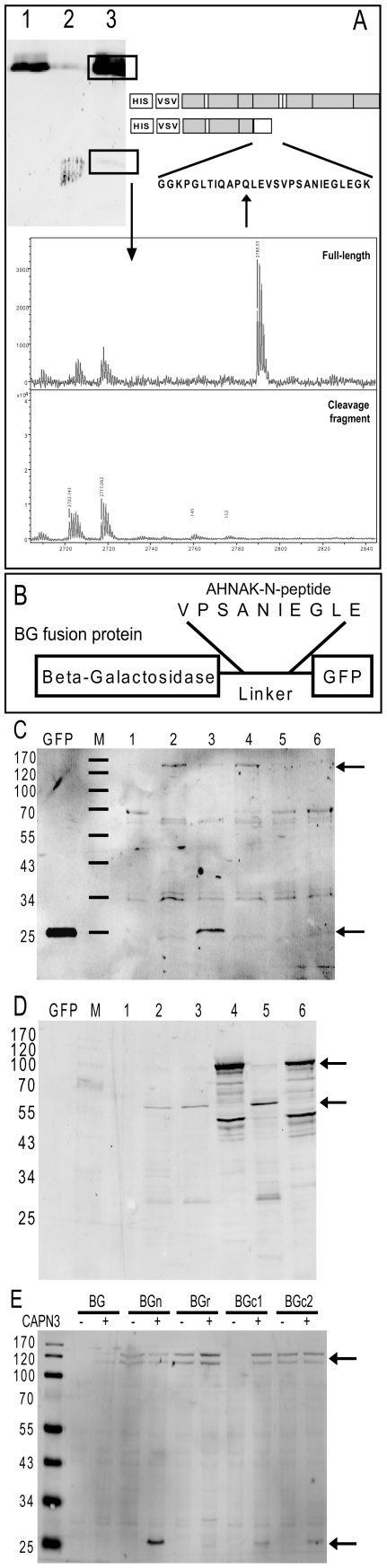
A peptide cleavage motif identifed in CAPN3. A) Cells were transiently co-transfected with a HIS-VSV tagged AHNAK-N domain and CAPN3 (active) or CAPN3^C129S^ (inactive). Cells were lysed and analyzed on western blot for cleavage with a VSV antibody. Lane1 = AHNAK-N+CAPN3^C129S^, Lane2 = AHNAK-N, Lane3 = AHNAK-N+CAPN3. Separately, HIS-tagged AHNAK proteins were purified and isolated from Coomassie Blue stained gels as indicated with the boxed bands, and analyzed with LC-MS. The spectrum depicts the most N-terminal peptide that was only identified in full-length AHNAK-N, and is therefore likely at the site of cleavage. B) Schematic representation of the β-Galactosidase-GFP (BG) fusion protein with the AHNAK-N peptide motif introduced into the linker region. C+D) BG fusion constructs with or without AHNAK-N peptide were co-expressed with active or inactive CAPN3, and cells were analyzed on western blot for cleavage with specific antibodies for GFP (C) or CAPN3 (D). 1 = non-transfected, 2 = BG+CAPN3, 3 = BGpeptide+CAPN3, 4 = BGpeptide+C129S, 5 = CAPN3, 6 = C129S. Arrows denote uncleaved (120 kDa) and cleaved (25 kDa) BG fusion protein (C) or inactive (94 kDa) and active (55 kDa) CAPN3 (D). E) as in C), but now the cleavage motifs identified in AHNAK-N domain, AHNAK-C1 and AHNAK-C2 domains are analyzed in the BG fusion protein, as is a homologous peptides found in the AHNAK-Repeat domain, which does not conform to the motif. + and – refer to active and inactive CAPN3 respectively. All cleavage experiments were performed in HEK-293T and MCF7 cells, with comparable results.

Upper part: List of described CAPN3 substrates in which the putative CAPN3 cleavage motif was identified. For each substrate the peptide motif is shown, with key residues spaced and underlined. The peptides were tested for cleavage potential in a BG fusion protein and judged as cleaved (+), partially cleaved (+/−), or not cleaved (−). In Figure 1F BGn (with peptide from AHNAK-N) is a good (+) substrate, BGc1 and -c2 are moderate (+/−), and BGr is bad (−). In addition, the protein and gene ID are given, the location of the motif, and the literature reference. Titin_952–1540_ and AHNAK-R were described as non-substrates and contain no motif sequence. For AHNAK-R a peptide with Blast homology to the peptide from AHNAK-N is shown, with in bold the residue that is not conform the cleavage motif. Middle part: Single mutants of the AHNAK-N and FLNC peptides were analyzed for cleavage potential. Mutated residues are shown in bold. Lower part: Several candidate peptides conforming to the general motif or the specific motif were analyzed for cleavage potential. Most peptides were also tested in a reverse orientation of the fusion proteins (GB, N-terminal GFP and C-terminal β-Galactosidase), never to give a discrepant result. Representative western blots for table 1 are included in Figure S1.

The position of this motif is in good agreement with estimated fragment sizes from published western blots. We used the first substrate in our motif screen, AHNAK, to experimentally confirm that cleavage occurs within close vicinity of the motif. The HIS-tagged N-terminal domain of AHNAK was co-expressed with CAPN3 as described previously [17] and the AHNAK protein fragments were purified by means of their HIS6-tag, resolved on SDS-PAGE gels, and subjected to mass spectrometry analysis. The peptide nearest to the HIS6-tag that was solely indentified in the full-length protein (Figure 1A) contained the predicted cleavage sequence, showing that the motif is at the site of cleavage.

We next asked whether this motif contains all of the essential information for CAPN3 mediated cleavage. To investigate this we used a fusion protein (BG) consisting of an N-terminal β-Galactosidase and a C-terminal GFP connected by a flexible linker (Figure 1B). The 10 amino acid sequence identified in AHNAK-N was inserted into the linker (Figure 1B), and fusion constructs with and without the AHNAK motif were co-expressed with CAPN3 or its proteolytically inactive mutant CAPN3^C129S^. [3] Figure 1C shows that CAPN3 is able to cleave the fusion protein only when it contains the AHNAK peptide, while CAPN3^C129S^ is unable to do so. This indicates that the AHNAK-N peptide contains all of the essential information for CAPN3 mediated cleavage. Correct CAPN3 expression was confirmed by western blot analysis using a CAPN3-specific antibody (Figure 1D).

We further verified that CAPN3 recognizes the motif. We proceeded by introducing the two additional predicted AHNAK peptides into our fusion protein and found that they were also cleaved upon co-expression with CAPN3 (Figure 1E), consistent with previous data that CAPN3 cleaves AHNAK at multiple sites. [17] One AHNAK peptide sequence identified by Blast similarity to the confirmed AHNAK-N peptide however was not cleaved (Figure 1E). Interestingly, this peptide maps to an AHNAK domain that was previously excluded to serve as a substrate for CAPN3 [17] and deviates from the motif only at position 9 where it contains a proline instead of [LIMV]. From this we conclude that CAPN3 indeed recognizes a primary sequence motif.

### Assignment of four key residues

The remaining peptides from the described substrates were all cleaved by CAPN3 in our assay (Table 1, Figure S1). As mentioned before the motif consists of a backbone and 4 key residues. We selected two peptides (the AHNAK peptide and the predicted Filamin C (FLNC, MIM*102565) peptide) based on their most dissimilar backbone residues and subjected them to extensive mutational analysis (Table 1). Mutation of positions 1 and 10 (key residues 1 and 4) to alanine abolishes cleavage. Alanine mutation of position 9 (key residue 3) has no strong effect. However, introducing a proline at this position (a mutation that mimics the Blast similarity result of Figure 1E) prevents cleavage, whereas a proline insertion at non-key residue position 2 does not affect cleavage. From the naturally occurring peptides in Table 1 it can be inferred that in the backbone of the motif almost every combination of amino acids is allowed. From this analysis we conclude that the 10 amino acids spanning CAPN3 cleavage motif consist of 4 key residues: amino acids 1 and 10 are essential, where 5 and 9 are of only moderate importance. It appears that the span width of the motif (10 aa) can be relatively variable, as backbone deletions do not always impair cleavage efficiency (Table 1). This information led to the identification of a putative cleavage site in AHNAK (AHNAK-C2.2), which had been described[17] and indeed was cleaved in our assay (Table 1).

### Predicting new CAPN3 substrates

All previous studies focused on known substrates, whereas our ultimate goal was to capture the general identifier of CAPN3 substrates and so to predict and verify the Calpainome of CAPN3. We therefore screened the motif against the UniProt database and retrieved 8,181 protein hits in human alone. While not entirely impossible (CAPN3 is hypothesized to cleave virtually everything within its vicinity due to its short half-life)[5] this seems unlikely and probably other constraints apply; an obvious one being that CAPN3 is muscle specific. We tested several candidate peptides based on their co-occurence with CAPN3 or myopathy in PubMed abstracts, and found some of them to be cleaved by CAPN3 (Table 1) confirming the predictive potential of the motif. Some peptides however did not show cleavage (see Discussion).

To generate a more specific list of putative substrates we created a more stringent motif by including information about the backbone residues. We added the amino acid identities for each of the 10 positions for the best performing peptides in our cleavage assay (see Methods section). This motif will therefore not represent all CAPN3 substrates, but does leave us with a more workable subset of potential substrates. The stringent motif ([VLM][PGED][SAGEKL] putX(0,1)[VAIML][ESPQLK][GKEY][VLA][ED]) predicts 325 substrates in human (Table S1). We selected a subset of substrate peptides representative for the functional diversity of the 325 proteins and which share less then 50% sequence identity to the peptides used to construct the motif. These peptides were tested positive in the BG fusion protein assay with a success rate of 88% (14/16 peptides: Table 1).

We next investigated if the presentation of the motif in the native proteins might allow for recognition by CAPN3. For 18 predicted substrates the crystal structure had been resolved, showing clear surface presentation of the motif in all cases (Table S1). Moreover, secondary structure predictions suggest that 67% of the cleavage sites are located in relatively non-structured protein segments, which is consistent with predictions for Calpain 1 and 2. [13] (data not shown) This indicates that the motif adopts a tertiary conformation and is presented in a flexible loop, consistent with the large backbone of the motif. [9] Finally, the structure of the described substrate FLNC is predicted to fit into the active site of CAPN3 in a docking experiment (Figure S2). Though not a formal proof, these results support our biochemical experiments and predict that the peptide motif can be recognized by CAPN3 in its natural context.

To confirm that the restricted motif yields biologically relevant candidate substrates we performed multiple biosemantic analyses. As CAPN3 is muscle-specific we can only analyze co-expression in muscle, which is less informative than when comparing expression in multiple tissues. To overcome this limitation, we did a concept analysis based on MedLine abstracts [19] to confirm that the restricted motif yields relevant predictions based on co-occurrence in the biomedical literature. [19] Figure S3A shows the concepts that are most frequently found to be associated with our putative 325 substrates. The top ranked concept is Cytoskeletal Proteins, consistent with the hypothesis that CAPN3 is a cytoskeleton remodeler. In the right panel of Figure S3A we listed those proteins that associate most strongly with the concept cytoskeleton. Only 2 of the 29 associated putative substrates were used in the definition of the cleavage motif, the others are new cytoskeletal CAPN3 substrate candidates. Other concepts associated with the 325 putative substrates relate to cell architecture and open the possibility for a role of CAPN3 in mitosis. The latter is interesting as a recent study reported on a population of CAPN3 expressing myogenic reserve cells in which CAPN3 indirectly regulated MyoD. [20] We next grouped all 325 proteins based on conceptual overlap before annotating with concepts, and plotted the results in a heat map (Figure S3B, Table S2). This gives a better view of which type of proteins can be found in the list. Again, we find several clusters of structural proteins. Other proteins in the list appear to participate in different biological processes such as mitosis, vesicle trafficking, apoptosis, transcription, calcium transport. For several of these processes some indication of CAPN3 involvement can be found in literature. [21]–[24] In conclusion, based on MedLine abstracts the putative substrates are strongly associated with the cytoskeleton. Additional GO term pathway annotation analysis (Figure S3C, Table S3) shows the strongest overrepresentation with GO terms that relate to cell structure and cyto-architecture. This corroborates on the hypothesis of CAPN3 being a cytoskeleton remodeler and strengthens our belief that we have identified a genuine CAPN3 cleavage motif.

**Figure 2:**
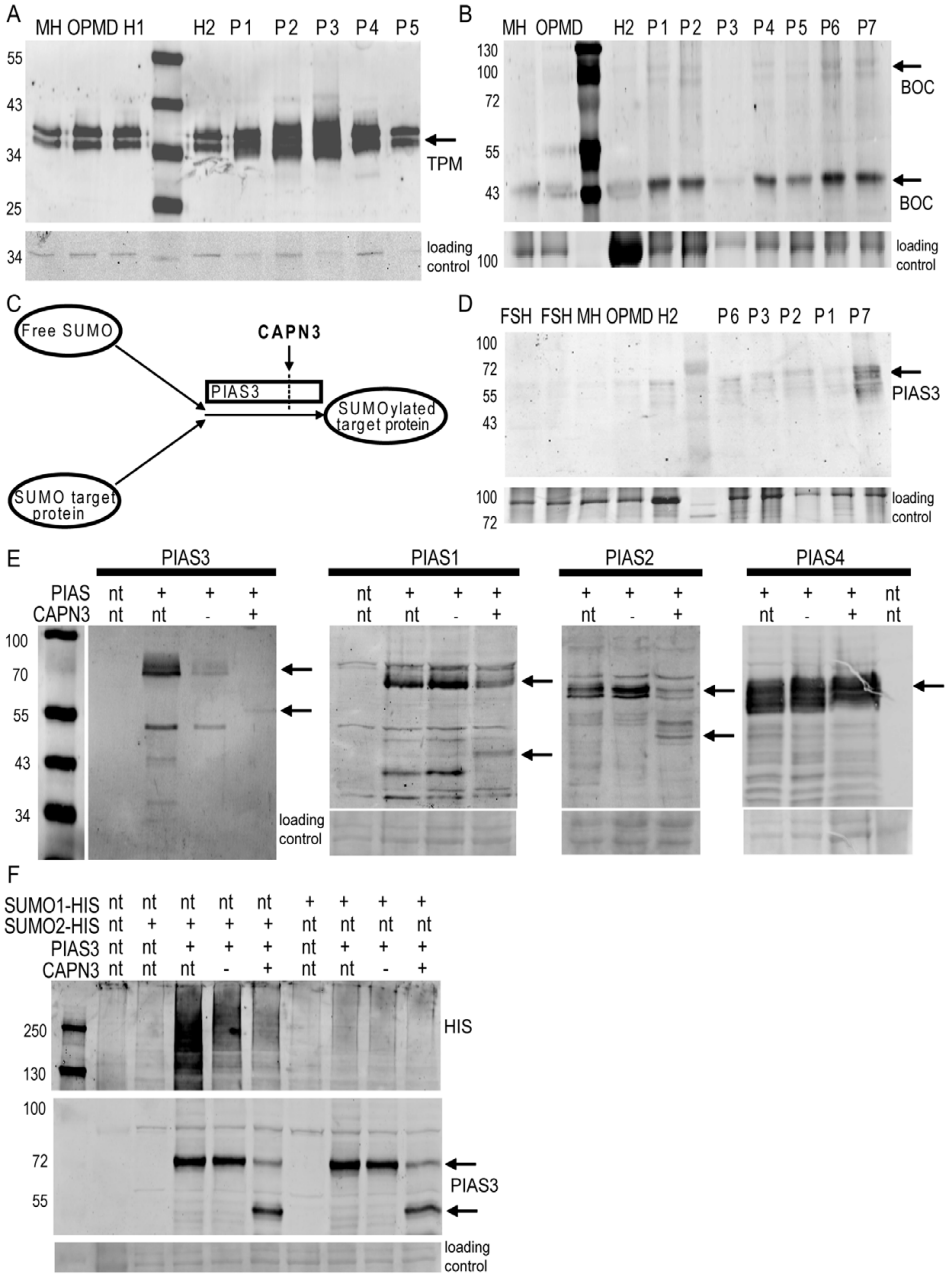
Verification of putative CAPN3 targets. A,B,D) Skeletal muscle homogenates of 5–7 Calpainopathy patients (P1-7), 1–2 healthy controls (H1-2) and 2–4 unrelated muscular dystrophies (OPMD, MH, FSHD) were analyzed on western blot with specific antibodies for Tropomyosin (A, at 35 kDa), BOC (B, at 110 and 45 kDa) and PIAS3 (D, at 60 kDa). The arrows denote the detected protein bands. C) Schematic model of PIAS3 E3 SUMO ligase activity, regulated by CAPN3. PIAS3 is involved in the covalent conjugation of Small Ubiquitin Like Modifier (SUMO) proteins to target proteins. PIAS3 has a CAPN3 cleavage motif at the C-terminus. E) FLAG tagged PIAS1, 2, 3, and 4 were transiently expressed with active or inactive CAPN3, and cleavage was analyzed on western blot with a FLAG specific antibody. Upon co-expression with active CAPN3 PIAS3, PIAS1 and PIAS2 are cleaved at the C-terminus as predicted. PIAS4 is not cleaved. The arrows denote uncleaved and cleaved FLAG-PIAS. Nt means not transfected, + and – refer to active and inactive CAPN3 respectively. F) PIAS3 was transiently expressed in an in vivo sumoylation assay with HIS6-SUMO1 or HIS6-SUMO2 and active or inactive CAPN3. Cells were lysed directly in sample buffer and sumoylation levels were estimated on western blot with a HIS antibody (upper panel). Correct expression and CAPN3 mediated cleavage was confirmed with a FLAG antibody (lower panel).

However, this does not prove that all 325 proteins are *in vivo* substrates. We therefore proceeded with studying muscle protein homogenates from Calpainopathy patients for their levels of two predicted substrates that represent some of the functional diversity of the predicted substrates: the cytoskeletal protein Tropomyosin (TPM2, MIM*190990), and the myogenic differentiation protein Brother of CDO precursor (BOC, MIM*608708). Figure 2 shows an accumulation of Tropomyosin (Figure 2A) and BOC (Figure 2B) in skeletal muscle of Calpainopathy patients compared to healthy and disease controls. Thus, based on our elucidation of the CAPN3 cleavage motif we find explainable disturbances in the levels of two predicted substrates in patients.

We selected one predicted substrate, PIAS3 (MIM*605987), for further inquiry, because it has two homologous family members that contain the general motif (PIAS1 and PIAS2) and a third family member without motif (PIAS4). PIAS3 is an ubiquitously expressed E3 SUMO ligase (Figure 2C) implicated in many signaling pathways including NFκB[25], which is perturbed in LGMD2A. [22] We first verified the levels of PIAS3 in CAPN3 deficient muscle sections (Figure 2D). This provided evidence for a slight increase in Calpainopathy tissue, compared to disease controls. To confirm that PIAS3 can be cleaved by CAPN3 we co-expressed FLAG-tagged PIAS3 with CAPN3 and observed a cleavage fragment of the predicted size (Figure 2E). PIAS1 and PIAS2, which contain the general CAPN3 cleavage motif, are similarly cleaved (Figure 2E). However, PIAS4, which has neither motif, is not cleaved (Figure 2E), showing that our motif can not only predict new targets but can also differentiate between homologous family-members based on potential substrate properties.

As mentioned above PIAS3 has E3 SUMO ligase activity (Figure 2C), which we expected to be regulated by CAPN3 proteolysis. When we co-expressed HIS6-tagged SUMO2 with PIAS3 and CAPN3 in an *in vivo* sumoylation assay, cleavage of PIAS3 impaired its sumoylation activity (Figure 2F). We confirmed our observation with a HIS6 pull-down (Figure S4), proving that this loss of signal involves covalently conjugated SUMO2 moieties. Furthermore, PIAS3 has the capacity to autosumoylate and CAPN3 cleavage completely abolishes this auto-sumoylation (Figure S4), thus negatively regulating PIAS3 activity.

Based on these results we were interested to see whether PIAS activity can be detected in skeletal muscle sections. We performed an immunofluorescent staining for SUMO2 in muscle cryosections of two patients with highest PIAS3 levels (P2 and P7 in Figure 2D) (Figure S5), hypothesizing that in absence of CAPN3 the PIAS proteins are not cleaved and therefore more active, resulting in higher sumoylation. In healthy control tissue distinct nuclear speckles (possibly PML bodies[26]) are visible in non-muscle cells. In muscle nuclei these speckles cannot be detected. In the CAPN3 deficient muscle sections however, nuclear speckle staining can also be observed in myonuclei, indicating increased sumoylation in muscle cells of these patients (Figure S5). Three disease controls also failed to show SUMO2 staining in muscle. Taken together this provides strong indication that CAPN3 regulates PIAS3 SUMO ligase activity in skeletal muscle.

## Discussion

We have provided evidence that CAPN3 recognizes a primary sequence motif in its substrates. We observed that peptides from confirmed CAPN3 substrates contain the information for CAPN3 mediated cleavage and can transform a non-substrate into a substrate. These peptides conform to a general motif of 4 key residues that is found in many proteins, including all previously reported substrates. Thus we provide a first explanation for why CAPN3 cleaves these proteins. The second, more stringent motif performs well in our biochemical assay. This is partly due to an introduced bias based on resemblance to well performing peptides. However, bioinformatics analysis of the putative substrates derived from this motif highlights plausible substrate classes such as apoptosis and vesicle trafficking, substantiating the importance of CAPN3 as a broad, yet specific regulator of cytoskeletal architecture. With the motif new substrates can be efficiently predicted, as shown by the increase in TPM and BOC levels in Calpainopathy patients and the regulation of PIAS proteins by CAPN3.

We observed that molecular modeling experiments predict that the peptide motif could be recognized by CAPN3. The mutation analysis we performed for our motif might have a different outcome when conducted within the folds of a different protein. Nevertheless, cleavage of mouse Cyclin A (*CCNA1*, MIM*604036, protein ID Q61456) by CAPN3 has been described. [27] The authors show that when a small peptide (residue 120–130) is deleted from this protein, cleavage is lost. Although this protein was not included in our original screen it harbors a putative cleavage site conforming to the first, general motif at residues 112–121. This is fully consistent with the motif being targeted by CAPN3 in its native protein environment.

For this study we did not consider actual determined cleavage sites. Most of these are autolytic with cleavage taking place intramolecularly within CAPN3, and their presentation could be different from intermolecular cleavage. When comparing the autolytic sites to the motif we found that all identified autolytic sites strongly resemble the first motif ([LIMV]X_(2–4)_[LIMV][DEQ]). We did find another peptide within CAPN3 that was functional in our biochemical system and may represent a new autolytic cleavage site. Intriguingly, 4 pathogenic amino acid substitutions have been described that affect this peptide (www.dmd.nl/capn3). Two mutations directly involve designated key residues, whereas the other two target amino acids flanking key residues. Two of these mutations show abnormal CAPN3 signal on western blot [28], [29], suggesting that these mutations may interfere with normal CAPN3 autoregulation; a mechanism that was also recently suggested by Garnham et al. [30]

The first step in the autolytic activation of CAPN3 is cleavage of the internal IS1 sequence. It is interesting that the CAPN3 IS1 sequence contains a peptide that conforms to our first, general motif. Diaz *et al* use the proteolytic core to show that IS1 is flanked by two flexible autolytic sites. [31] Moreover, they model the IS1 sequence as an α-helix (secondary structure). Introducing a proline mutation to disrupt this putative helix drastically increases the autolytic properties of IS1. This is consistent with a preference of the active core for flexible tertiary folds. We tested the IS1 peptide in our system and could strongly increase its cleavage potential by introducing a glycine at position 7 to increase flexibility (Table 1).

With our stringent motif we have predicted 325 CAPN3 substrates, whereas the first motif predicted several thousands of substrates. This raises the question as to how specific CAPN3 cleavage is. The first motif is a common feature of many proteins and suggests that the chance of CAPN3 encountering a substrate is extremely high. CAPN3 activity would then be limited by its means of activation and inactivation only. A protease that recognizes thousands of substrates is potentially incredibly destructive and needs a tight regulation of its own activity. This would explain why CAPN3 protein is rapidly lost upon activation, as was recently also suggested by Garnham *et al* [30]. The first motif resembles the IS1 sequence, which acts as an inhibitory peptide. [31] Loss of this peptide leaves the active site cavity open for IS1 resembling structures; flexible non-structural protein loops [9] with similarities to the inhibitory IS1 sequence that would fit into the cavity. Among the peptides that fit are multiple sequences of CAPN3 itself. Opening of the cavity not only results in activation, but is rapidly followed by progressive autolysis and loss of the protease. [8], [32] Thus, both inhibition and inactivation are self-regulated and CAPN3 activity is local by default, which corroborates the previous model by Beckmann and Spencer [5]. This mechanism seems to plausibly reconcile the apparent contradiction of a highly reactive protease, which yet recognizes potentially thousands of protein substrates (Figure S6).

It is unclear whether the basic, more relaxed motif is sufficient under all circumstances and many predicted targets will never encounter the muscle-specific CAPN3. Moreover, the sarcomere-bound form of CAPN3 is heavily restricted in its substrate encounters due to a low diffusion rate. [5] CAPN3 −/− mice display misaligned sarcomeres[33] and cannot undergo cycles of degeneration and regeneration indicating CAPN3 as an essential muscle remodeling protein. [34] Muscle has a high demand for focal remodeling (exercise, repair, growth, etc) and would benefit greatly from a localized unidirectional molecular remodeling switch. So a highly reactive broad-spectrum protease that is specific, and which is able to switch on and off irreversibly by itself, in a short time span, is a plausible remodeling agent in such a tissue.

Previous mutation analyses indicated that most pathogenic substitutions in CAPN3 either influence its activity directly or indirectly by modifying its autolytic properties. [30] The broad motif makes it unlikely that different CAPN3 mutations cause an LGMD phenotype due to the deregulation of only one or two substrates. Still it will be interesting to see whether mutations in cleavage sites have been described for the uncovered substrates, and with which phenotypic consequences. Of special interest is PIAS3, which is a target for both CAPN3 and TRIM32 (MIM*602290). [35] TRIM32 mutations cause LGMD2H (OMIM#254110), a phenotype similar to LGMD2A. [36] Therefore PIAS regulation may connect two genetically distinct types of LGMD.

It will be interesting to see whether our approach would also work for other Calpain family members and compare their predicted cleavage motifs, and substrate specificities. In addition, methods are available [37] which may be used to improve the predictive potential of our motif. While the folding of proteins has long eluded the mechanism behind protein substrate recognition, our studies show that molecular and data mining tools have improved much, putting a comprehension of proteolytic cleavage within reach.

## Materials and Methods

### Ethics statement

Patient biopsies were acquired through Dr. R Charlton, Freeman Hospital, Newcastle-upon-Tyne, UK. All analyzed patients had a confirmed molecular diagnosis, and met ethical criteria for inclusion within the study including written informed consent. All patients were recruited at Newcastle University, according to the ethics committee there. Those biopsies that met the criteria for inclusion in our study were send to us. Thus, no patients were recruited specifically for our studies.

### Patient mutation analysis

The unrelated muscular dystrophy cases were all genetically confirmed and include patients with OPMD (1), MH (1) and FSHD (2). LGMD2A patients had the following genetic mutations: P1) X01:c.145C>T and X11:c.1468 C>T, P2) X02:c.327_328delCC and X11:c.1477C>T, P3) X02: c.327_328dupCC and X11:c.1435A>G, P4) X05:c.759_761delGAA and X11:c.1468C>T, P5) X04:c.550delA and X11:c.1435A>G. P6) X05:c.633G>C and X10:c.1319G>A. P7) X22:c.2314_2317delGACA. P3, P5 and P7 display a complete absence of CAPN3 protein. P1, P2 and P6 have reduced CAPN3 protein levels. In P4 CAPN3 levels are normal.

### Antibodies

RαGFP (1;5,000 Abcam), GαR-IRD680 and GαM-IRD800 (both 1;5,000 Westburg), MαCAPN3 NCL-12A2 (1;100 Novocastra), MαTPM TM311 (1;2,000 Sigma), MaBOC (1;1,000 R&D), RaPIAS3 (1;1,000 Santa Cruz), MaVSV P5D4 (1;5,000), RaFLAG (1;2,000 Sigma), MaHIS6 (1;2,000 Cell signaling), RaSUMO2/3 (1;5,000 gift from dr. A.C. Vertegaal, Leiden, The Netherlands), MaDMD NCL-DYS2 (1;10 Novocastra), GaM488 and GaR594 (1;750 and 1;2,000 Molecular Probes).

### Cell culture

HEK293T [38] and MCF7 [38] cells were cultured in Dulbecco's modified Eagle's medium supplemented with Glucose, Pen/Strep 10% FCS and Glutamax (all Gibco-BRL) and grown at 37°C and 5% CO2.

### DNA cloning and transfection

The β-Galactosidase-GFP (BG) and GFP- β-Galactosidase (GB) vectors were a gift from Dr. JM Daniel, West Hamilton, Canada. Target sequences were cloned by ligating pre-synthesized complementary primers (Invitrogen). Primer sequences available on request. All constructs were sequence verified (LGTC, Leiden, The Netherlands). CAPN3 in pCDNA4 was a gift from Dr. K Bushby, Newcastle, UK. The C129S mutant was constructed by site directed mutagenesis and confirmed with direct sequencing (LGTC). Expression vectors for HIS6-SUMO and FLAG-PIAS were a gift from G. Roukens, LUMC, The Netherlands. DNA was transfected with JET-PEI (Poly-Plus) according to manufacturers protocol. 48 h post-transfection cells were washed and lysed in sample buffer.

### Protein purification and mass spectrometry

Transiently transfected cells were lysed in ice cold lysis buffer (50 mM Tris, pH7.4, 0.15M NaCl, 0.2% Triton X-100, 2× protease inhibitor cocktail (Roche)) and spun down 30 min, 4°C. TALON resin (Clontech) was washed, pre-equilibrated and added for 2 h 4°C, and subsequently spun down at 700 g, 4°C. The resin was washed 3x and loaded on a column. A 300 bed volume wash was followed by elution in 200 mM imidazole. Purified AHNAK fragments were separated on SDS-PAGE gels and Coomassie Blue stained bands in-gel digested and analyzed with LC-MS. Fingerprints were screened with the MASCOT search engine.

SDS-PAGE, immunoblotting and immunohistochemistry were performed as described before. [17] Coomassie Blue staining of the gel after transfer was carried out to confirm equal loading.

### Sequence analysis

Sequence analysis was done with web-based tools (www.expasy.org). Protein sequences of described CAPN3 substrates were retrieved from Uniprot (release 14.6) (http://www.expasy.org/sprot/) and aligned in TEIRESIAS (http://cbcsrv.watson.ibm.com/Tspd.html). [18] The following parameters resulted in motif identification: Amino acids grouped according to chemical properties, the minimum number of typical residues (L) was set at 4, the maximum extent between two residues (W) was set at 10, and the minimum number of proteins in which a pattern should occur (K) was set at 10. The motif was [LIMV]X(4)[LIMV]X(2)[LIMV][DE]. (Or [LIMV]X(3,4)[LIMV]X(2)[LIMV][DE] with spacing variation.)

For the second motif the amino acids at each position for several cleaved peptides was “added”. As the first motif was thereby discarded we started with the predicted functional peptides. Adding of AHNAK2, CAPN3 and CAST gave: [LM][DPE][LSK][RFK][SK.][IMV][KPL][EKY][V][DE] with five protein hits. Including AHNAK-C2.2 gave: [VLM][DPGE][LSIK]X(1,2)[LIMV][KPL][EKY]V[DE]. Including the other AHNAK peptides for consistency and the functional mutants that were not conform the first motif gave: [VLM][DPGE][LAIGESK]X(1,2)[LIMVA][KESQPL][GEKY][LVA][DE]. Careful examination of all tested peptides revealed that glycine is present at site 4 only in dysfunctional peptides. This brings the final stringent motif to [VLM][PGED][SAGEKL]{G}X(0,1)[VAIML][ESPQLK][GKEY][VLA][ED] with 325 protein hits in human. On average the peptides used to construct the motif contributed 18% to the motif, indicating that the 325 predicted substrates were not identified based on a high level of sequence identity to the test set (jackknife cross-validation [39]). In statistical prediction, the following three test methods are often used to examine a predictor for its effectiveness in practical application: independent dataset test, subsampling (K-fold cross-validation) test, and jackknife test [40]. However, as elucidated by [39] and demonstrated by Eq.50 in [41], among the three cross-validation methods, the jackknife test is deemed the most objective that can always yield a unique result for a given benchmark dataset, and hence has been increasingly used and widely recognized by investigators to examine the accuracy of various predictors (see, e.g. [42]–[44]). Accordingly, in this study the jackknife test was also adopted to analyze the prediction quality.

The motifs were screened against the Swissprot database (vs14.6, 16 Dec 2008) using ScanProsite (http://www.expasy.org/tools/scanprosite/). [45]–[47] Default settings (no splice variants, no overlap, not greedy) were applied, with a taxonomy restriction for *Homo sapiens*.

### MedLine analyses

The list of 325 predicted target proteins was analyzed with Anni v2.0, a text mining tool that serves as an automated interface to the biomedical literature (http://biosemantics.org/anni/). 193 proteins had a concept profile, i.e. a statistically weighted summarization of the context in which the protein appears in MedLine abstracts. For the other proteins there was not sufficient information available in MedLine to construct a concept profile. Which concepts (coming from the Unified Medical Language System vocabulary (includes amongst others: disease, tissue, cellular location, GO terms) were most strongly associated with the proteins in the list was analyzed.

### GO term analysis

A Gene Ontology analysis was done on the set of 325 predicted proteins to find those GO terms that are most informative for the set. The information content of a GO term is defined as the negative log of the probability to find a subset of the proteins annotated to that GO term.


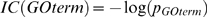
, where *p_GOterm_* is calculated using the hyper geometric distribution. Every GO category is evaluated individually. Biological process has 218, cellular component 239, and molecular function 238 proteins annotated in the root node respectively. The probability that a subset is annotated in the root node always results in a probability of 1, and therefore has an information content of 0. GO terms that are lower in the GO hierarchy will have a higher information content in general. Pathways were visualized with Matlab software.

### SUMO2 pull-down

Transiently transfected HEK-293T cells were lysed by scraping in 1 ml ice cold PBS with 2x protease inhibitor. An aliquot was used to measure protein content and verify expression on western blot. To the remaining lysate 3 ml denaturing buffer (6M Guanidium 0.1M Na2HPO4/NaH2PO4, 0.01M Tris/HCl, pH 8.0 plus 20 mM imidazole and 10 mM b-mercaptoethanol) was added. Preequilibrated NTA beads were incubated 4 h 4°C tumbling, spun down and washed. Subsequent washes with 8M urea (plus 0.1 M Na2HPO4/NaH2PO4, 0.01 M Tris/HCl, pH 8.0, 10 mM b-mercaptoethanol plus 0.2% Triton X-100) and 6M urea were followed by elution in 6M urea containing 200 mM imidazole.

### Structure analysis and docking

Secondary structure prediction was performed with two different webtools: PSIPRED [48], [49] and PHYRE [50].

The 3D structure of CAPN3 was obtained using homology modeling of Swiss-Model Server (vs 8.05). [51] The structure of CAPN2 (3DF0.pdb)[52] which shares 50% identity with CAPN3 was used as a model. (Calpain 4 and CAST structures were discarded.) The FLNC structure (2D7P.pdb) was downloaded from NCBI. Docking calculations were carried out with the program Bigger [53], [54], which requires as input the 3D structure for each of the two interacting proteins CAPN3 (Target) and FLNC (Probe). The structure of the complex is calculated through a soft docking approach. The algorithm generates populations of docked geometries with a maximal surface matching. The 500 model structures generated are ranked based on a computational function. The simulation was carried out without experimental restraints.

## Supporting Information

Figure S1 Summary of peptide analysis corresponding to Table 1. A) Multiple putative cleavage sequences from described substrates were cloned into the BG fusion protein and co-expressed with active (+) or inactive (−) CAPN3. Cells were analyzed on western blot with RaGFP. Results for Ezrin (EZR), Vinexin (SORBS3), and Talin (TLN1, 2 sites) are shown. Above the blot the motif is shown. B) The predicted key residues of the motif found in AHNAK-N were individually changed to alanine, cloned into the BG fusion protein and tested by co-expression with active or inactive CAPN3. Above the blot the AHNAK-N peptide sequence in the BG fusion protein is shown. C) As in B) but now for the peptide motif identified in FLNC. D) Several target proteins that derive from the second, stringent motif screen were tested in the BG fusion protein assay. Results for BOC, AP1B1, AP2B1 and CTCF are shown. The fusion protein with AHNAK-N peptide and its non-cleaved L9P mutant were used as controls. The stringent motif sequence is depicted above the blot. E) The assay yields comparable results in a GFP-BGal fusion protein. The mutations shown in panel B were cloned in the "reversed" fusion protein and tested by co-expression with active and inactive CAPN3. In all panels arrows denote the uncleaved and cleaved fusion protein.
(1.44 MB TIF)

Figure S2 FLNC is predicted to dock into the active site of CAPN3. A) We aligned the sequence of CAPN3 to that of Calpain 2 and modeled it onto the Calpain 2 structure, as done previously for CAPN3. [13] We imported this structure together with FLNC to a docking program (Bigger)[12] and allowed the software to dock FLNC (containing the putative cleavage motif) onto the CAPN3 structure without experimental restraints. The top 500 experimentally ranked docking solutions are shown. The structural model of CAPN3 is depicted in blue, with the active site in pink space fill. The dots represent the geometric center of FLNC in 500 docking solutions, ranked from red (high) to green (low) probability. No solution brings FLNC close to the active site of CAPN3. B) Same as in A) but the IS1 sequence of CAPN3, which blocks the active site, was removed to mimic proteolytic activation. Now, the docking solutions cluster towards the active site. C+D) Ribbon image of a representative solution from the top 10. The 10 highest ranked solutions present the motif within reach of the reactive cysteine. The modeled distances between the reactive cysteine and activating histidine of CAPN3 and the motif are (CAPN3-FLNC): C129-Y58 = 2.20Å, C129-E56 = 7.98Å, C129-E61 = 8.90Å, H333-E56 = 3.34 Å. For Calpain 2 a distance of 5Å is sufficient for cleavage. [52] Exchanging target (CAPN3) and probe (FLNC) resulted in the exact same top ranked solutions.
(9.43 MB TIF)

Figure S3 Data mining tools show conceptual overlap between CAPN3 and the list of putative substrates. A) The software program Anni was used to calculate concept profiles for all substrates. The concepts were scored and ranked according to occurrence. The concept "cytoskeletal proteins" is found most often. The proteins that associate with this concept are listed in the right column with in bold the proteins used in formation of the motif. B) All proteins were first clustered according to conceptual overlap, before annotating with concepts. Results were plotted in a heat map, showing distinct protein clusters (corresponding table in supplementary table S2). C) GO term analysis of the list of putative substrates (full table in supplementary table S3). GO term representation within the list was compared to the GO ontology database. The top 20 enriched hits for cellular component were analyzed with Matlab.
(2.36 MB TIF)

Figure S4 Regulation of PIAS3 sumoylase activity by CAPN3. A) In a SUMO2 pull down experiment the amount of SUMO2 conjugated proteins is decreased upon PIAS3 cleavage. HEK-293T cells were transfected with HIS6 tagged SUMO2, FLAG-PIAS3 and CAPN3 or CAPN3C129S, and cells were lysed 48 h post transfection in 6M Guanidium. SUMO2 conjugates were pulled down by means of the HIS6 tag with nickel NTA beads. Eluted proteins were analyzed for SUMO2 content on western blot with a SUMO2 specific antibody. + Means transfected, nt means non-transfected, - means transfected with inactive CAPN3C129S. B) A SUMO2 pull down experiment shows that PIAS3 autosumoylation is severely impaired upon CAPN3 mediated proteolytic cleavage. Cells were transfected as in A) and HIS6 tagged SUMO2 conjugates were pulled down with NTA beads. Pull down fractions were analyzed on western blot for FLAG-PIAS3 content with a FLAG specific antibody. Blots depict pulldown samples probed for FLAG-PIAS3 (upper panel) and pull down input lysates probed for FLAG-PIAS3 and CAPN3 (middle and bottom, respectively). Arrows denote sumoylated PIAS3, full-length and cleaved PIAS3, and inactive and active CAPN3.
(4.05 MB TIF)

Figure S5 SUMO2 is increased in myonuclei of LGMD2A patients. Single cryosections of skeletal muscle of LGMD2a patients P2 and P7, three healthy controls, and three disease controls (MH, OPMD, FSHD) were stained for Dystrophin (DMD, green, muscle membrane marker) and SUMO2 (red). Nuclei are stained with DAPI in blue. Distinct nuclear dots are seen in non-muscle nuclei (Arrowheads), and in LGMD2A myonuclei (Large arrows).
(4.59 MB TIF)

Figure S6 A model of CAPN3 function. Upon activation the chance that CAPN3 will encounter a substrate is enormous. The chance that the substrate is not CAPN3 is similarly high. However, as proteolysis proceeds and the number of non-processed substrates drops, the chance of CAPN3 encountering another CAPN3 increases. This will automatically control the number of active CAPN3 proteases inversely to the amount of processed substrates. Thus CAPN3 activity is local by default.
(0.77 MB TIF)

Table S1 325 predicted CAPN3 substrates. The specific motif sequence was screened against UniProt with the webtool ScanProsite, and yielded 325 unique proteins in human that contain the motif. For each of the 325 proteins the following information is listed: UniProt accession number, Protein ID, and Gene ID. For 18 of the proteins the crystal structure had been experimentally resolved. For these proteins a surface model of this structure in included below (hyperlink), with in red the cleavage motif.
(1.72 MB DOC)

Table S2 Conceptual clusters occurring in the list of putative CAPN3 substrates. The 325 putative substrates were loaded into the software program Anni and associated with concepts. The concepts associated with the 325 putative substrates were grouped into clusters and statistically weighed. The most significantly occurring clusters were annotated with concepts. The first column list the statistically weighed clusters, with in the second column the associated concepts. The third column shows the genes belonging to each cluster; with in bold those that tested positive in the fusion protein assay.
(0.29 MB DOC)

Table S3 GO term annotation analysis for the putative CAPN3 substrates. Gene Ontology pathway analysis was performed on the set of 325 putative substrates, to identity those pathways that are most informative for the set. The first column shows the entropy, where high entropy means a high content of information. The second column lists the calculated numbers (Substrates belonging to GO term | Absolute number of proteins belonging to GO term pathway | Total proteins associated with GO term | Total substrates in GO term pathway). In addition, the corresponding GO term ID, GO Pathway, and GO term are given in the last three columns. The top 20 hits for the Pathway Cellular Component are plotted in Figure S2c. The Table was cut at an entropy of 2.2669.
(0.13 MB DOC)
