# Supplementary figures and images for: Calpain 3 Is a Rapid-Action, Unidirectional Proteolytic Switch Central to Muscle Remodeling
Source: PLoS One. 2010 Aug 4;5(8):e11940. doi: 10.1371/journal.pone.0011940 (PMC2915920; doi:10.1371/journal.pone.0011940)

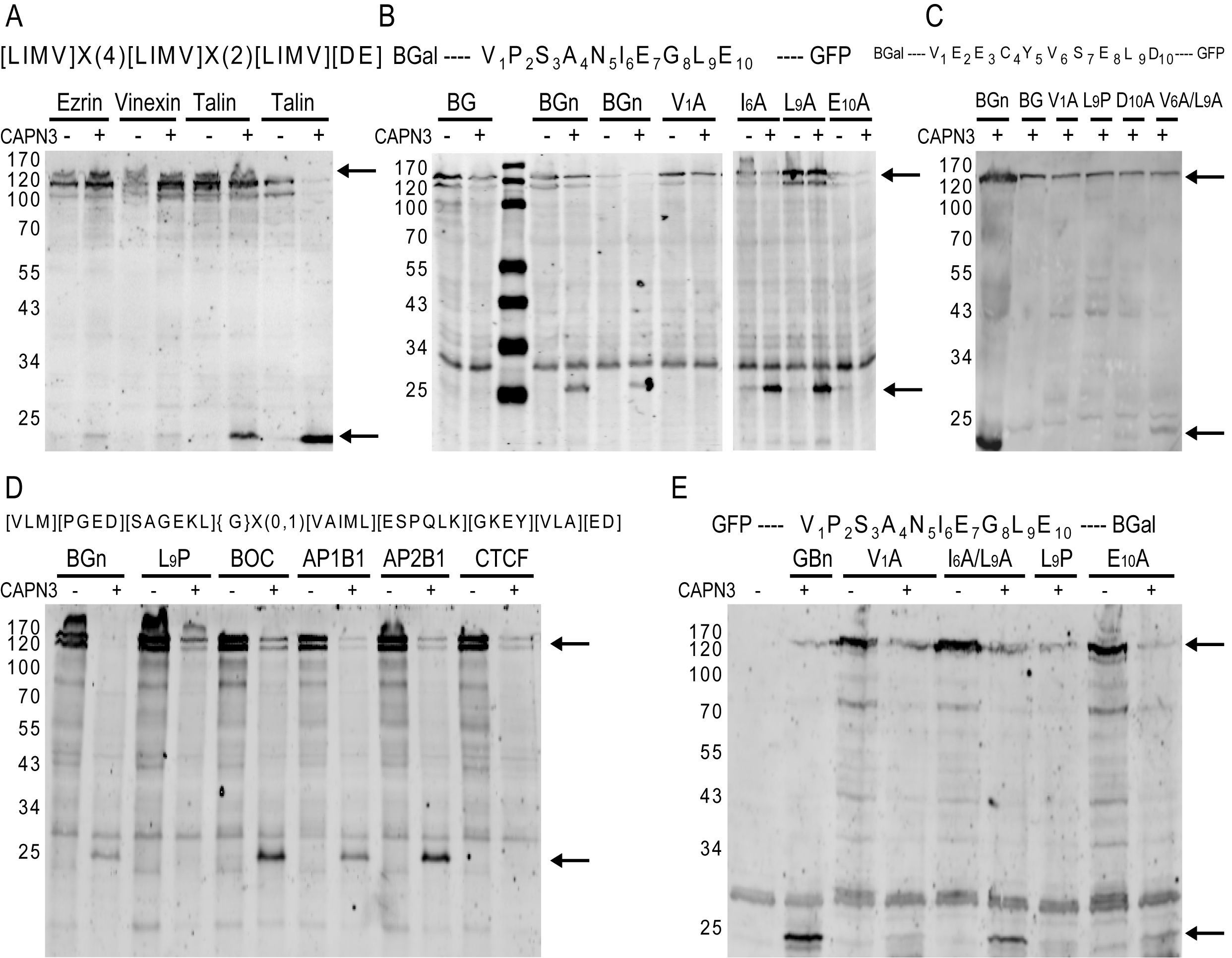

Supplement: Figure S1 — Summary of peptide analysis corresponding to Table 1. A) Multiple putative cleavage sequences from described substrates were cloned into the BG fusion protein and co-expressed with active (+) or inactive (−) CAPN3. Cells were analyzed on western blot with RaGFP. Results for Ezrin (EZR), Vinexin (SORBS3), and Talin (TLN1, 2 sites) are shown. Above the blot the motif is shown. B) The predicted key residues of the motif found in AHNAK-N were individually changed to alanine, cloned into the BG fusion protein and tested by co-expression with active or inactive CAPN3. Above the blot the AHNAK-N peptide sequence in the BG fusion protein is shown. C) As in B) but now for the peptide motif identified in FLNC. D) Several target proteins that derive from the second, stringent motif screen were tested in the BG fusion protein assay. Results for BOC, AP1B1, AP2B1 and CTCF are shown. The fusion protein with AHNAK-N peptide and its non-cleaved L9P mutant were used as controls. The stringent motif sequence is depicted above the blot. E) The assay yields comparable results in a GFP-BGal fusion protein. The mutations shown in panel B were cloned in the "reversed" fusion protein and tested by co-expression with active and inactive CAPN3. In all panels arrows denote the uncleaved and cleaved fusion protein. (1.44 MB TIF) [file pone.0011940.s001.tif]

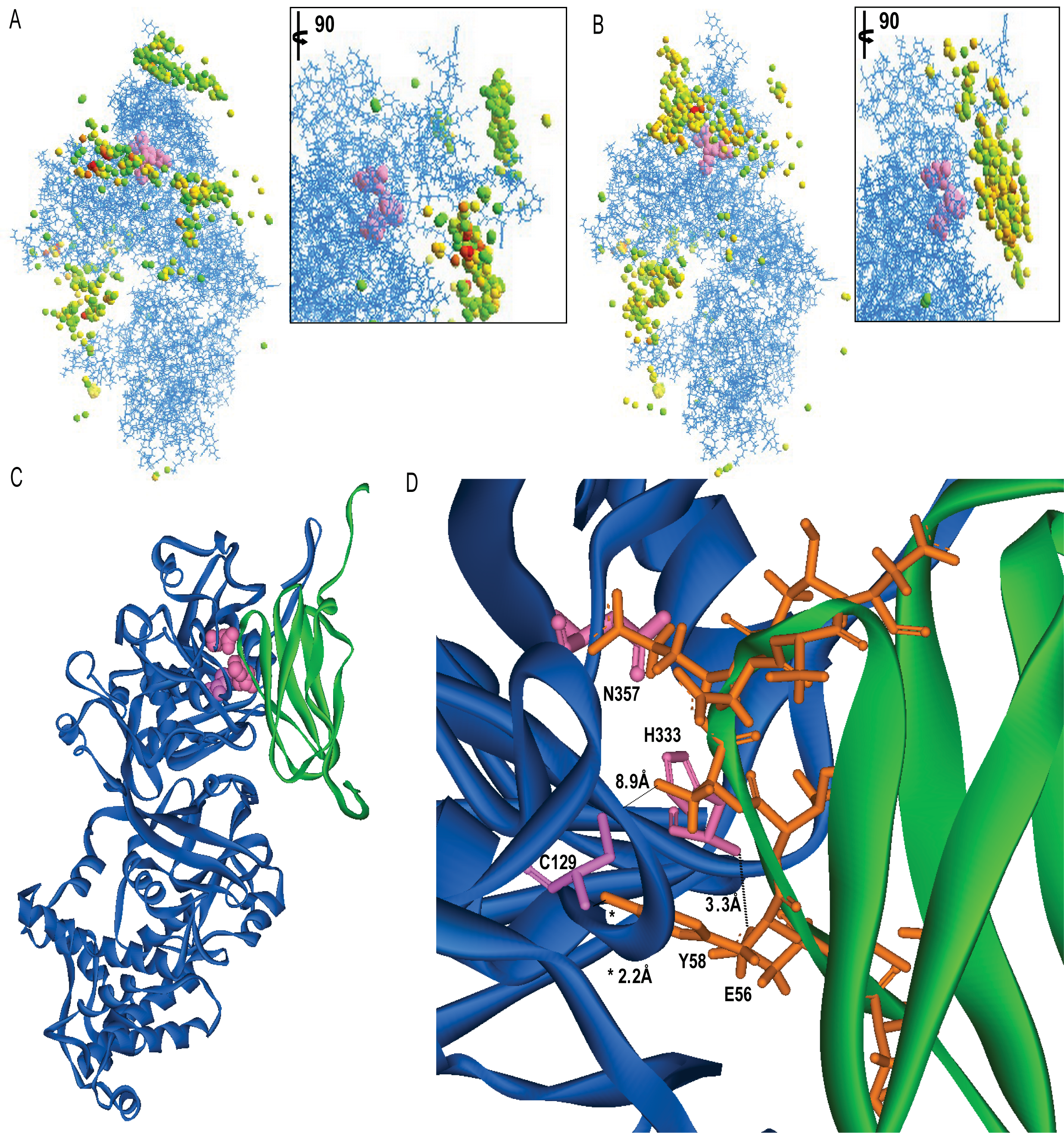

Supplement: Figure S2 — FLNC is predicted to dock into the active site of CAPN3. A) We aligned the sequence of CAPN3 to that of Calpain 2 and modeled it onto the Calpain 2 structure, as done previously for CAPN3. [13] We imported this structure together with FLNC to a docking program (Bigger)[12] and allowed the software to dock FLNC (containing the putative cleavage motif) onto the CAPN3 structure without experimental restraints. The top 500 experimentally ranked docking solutions are shown. The structural model of CAPN3 is depicted in blue, with the active site in pink space fill. The dots represent the geometric center of FLNC in 500 docking solutions, ranked from red (high) to green (low) probability. No solution brings FLNC close to the active site of CAPN3. B) Same as in A) but the IS1 sequence of CAPN3, which blocks the active site, was removed to mimic proteolytic activation. Now, the docking solutions cluster towards the active site. C+D) Ribbon image of a representative solution from the top 10. The 10 highest ranked solutions present the motif within reach of the reactive cysteine. The modeled distances between the reactive cysteine and activating histidine of CAPN3 and the motif are (CAPN3-FLNC): C129-Y58 = 2.20Å, C129-E56 = 7.98Å, C129-E61 = 8.90Å, H333-E56 = 3.34 Å. For Calpain 2 a distance of 5Å is sufficient for cleavage. [52] Exchanging target (CAPN3) and probe (FLNC) resulted in the exact same top ranked solutions. (9.43 MB TIF) [file pone.0011940.s002.tif]

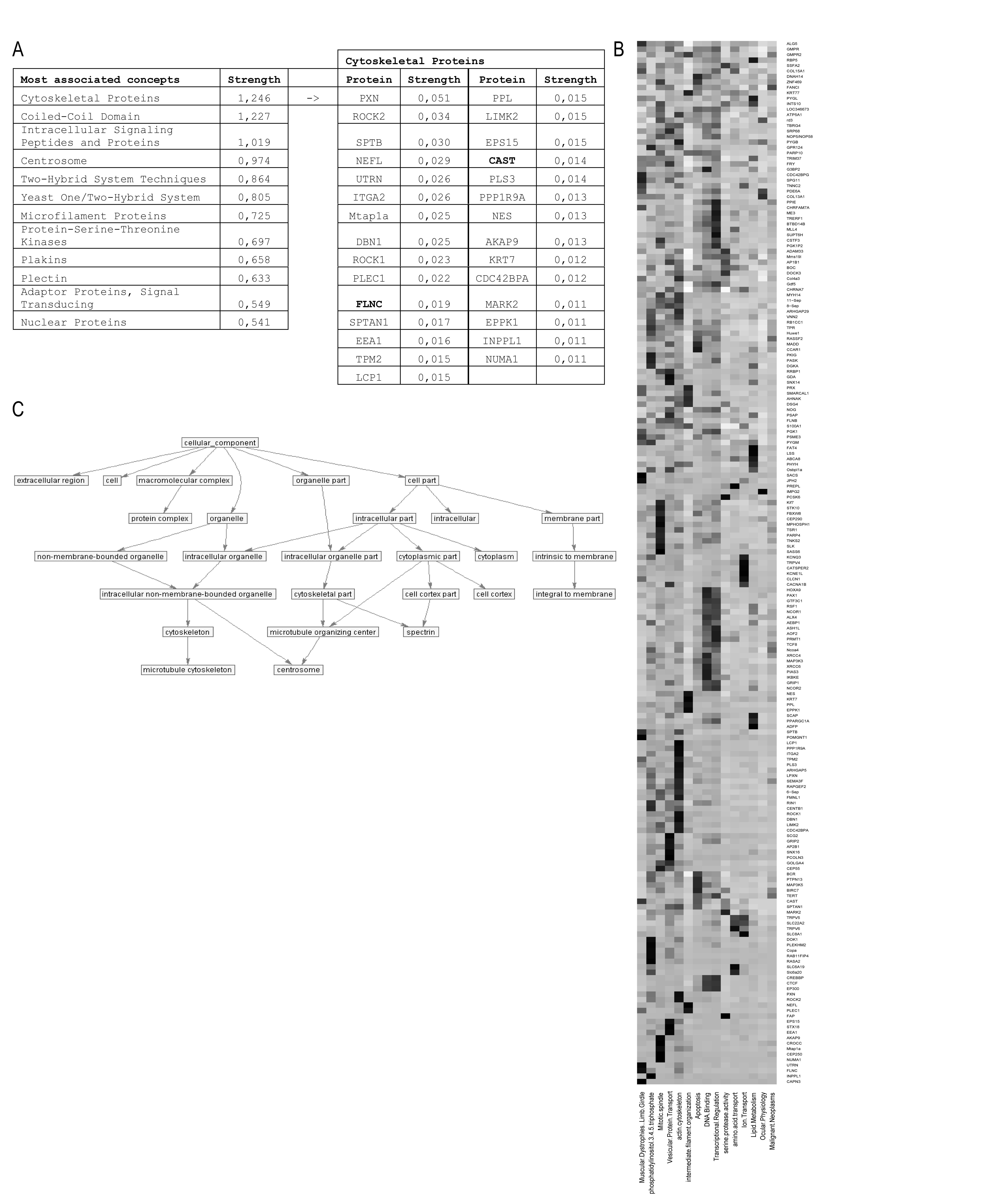

Supplement: Figure S3 — Data mining tools show conceptual overlap between CAPN3 and the list of putative substrates. A) The software program Anni was used to calculate concept profiles for all substrates. The concepts were scored and ranked according to occurrence. The concept "cytoskeletal proteins" is found most often. The proteins that associate with this concept are listed in the right column with in bold the proteins used in formation of the motif. B) All proteins were first clustered according to conceptual overlap, before annotating with concepts. Results were plotted in a heat map, showing distinct protein clusters (corresponding table in supplementary table S2). C) GO term analysis of the list of putative substrates (full table in supplementary table S3). GO term representation within the list was compared to the GO ontology database. The top 20 enriched hits for cellular component were analyzed with Matlab. (2.36 MB TIF) [file pone.0011940.s003.tif]

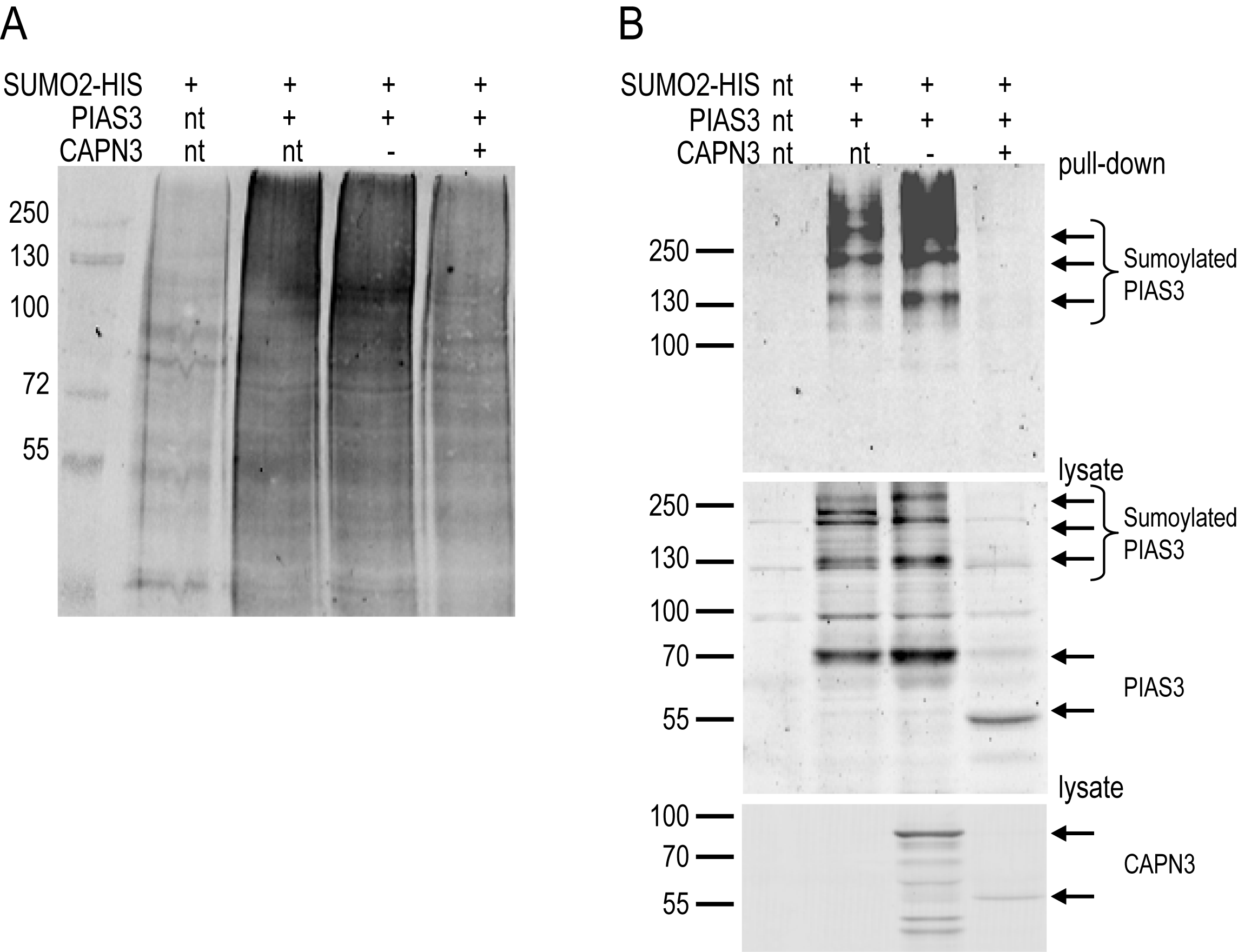

Supplement: Figure S4 — Regulation of PIAS3 sumoylase activity by CAPN3. A) In a SUMO2 pull down experiment the amount of SUMO2 conjugated proteins is decreased upon PIAS3 cleavage. HEK-293T cells were transfected with HIS6 tagged SUMO2, FLAG-PIAS3 and CAPN3 or CAPN3C129S, and cells were lysed 48 h post transfection in 6M Guanidium. SUMO2 conjugates were pulled down by means of the HIS6 tag with nickel NTA beads. Eluted proteins were analyzed for SUMO2 content on western blot with a SUMO2 specific antibody. + Means transfected, nt means non-transfected, - means transfected with inactive CAPN3C129S. B) A SUMO2 pull down experiment shows that PIAS3 autosumoylation is severely impaired upon CAPN3 mediated proteolytic cleavage. Cells were transfected as in A) and HIS6 tagged SUMO2 conjugates were pulled down with NTA beads. Pull down fractions were analyzed on western blot for FLAG-PIAS3 content with a FLAG specific antibody. Blots depict pulldown samples probed for FLAG-PIAS3 (upper panel) and pull down input lysates probed for FLAG-PIAS3 and CAPN3 (middle and bottom, respectively). Arrows denote sumoylated PIAS3, full-length and cleaved PIAS3, and inactive and active CAPN3. (4.05 MB TIF) [file pone.0011940.s004.tif]

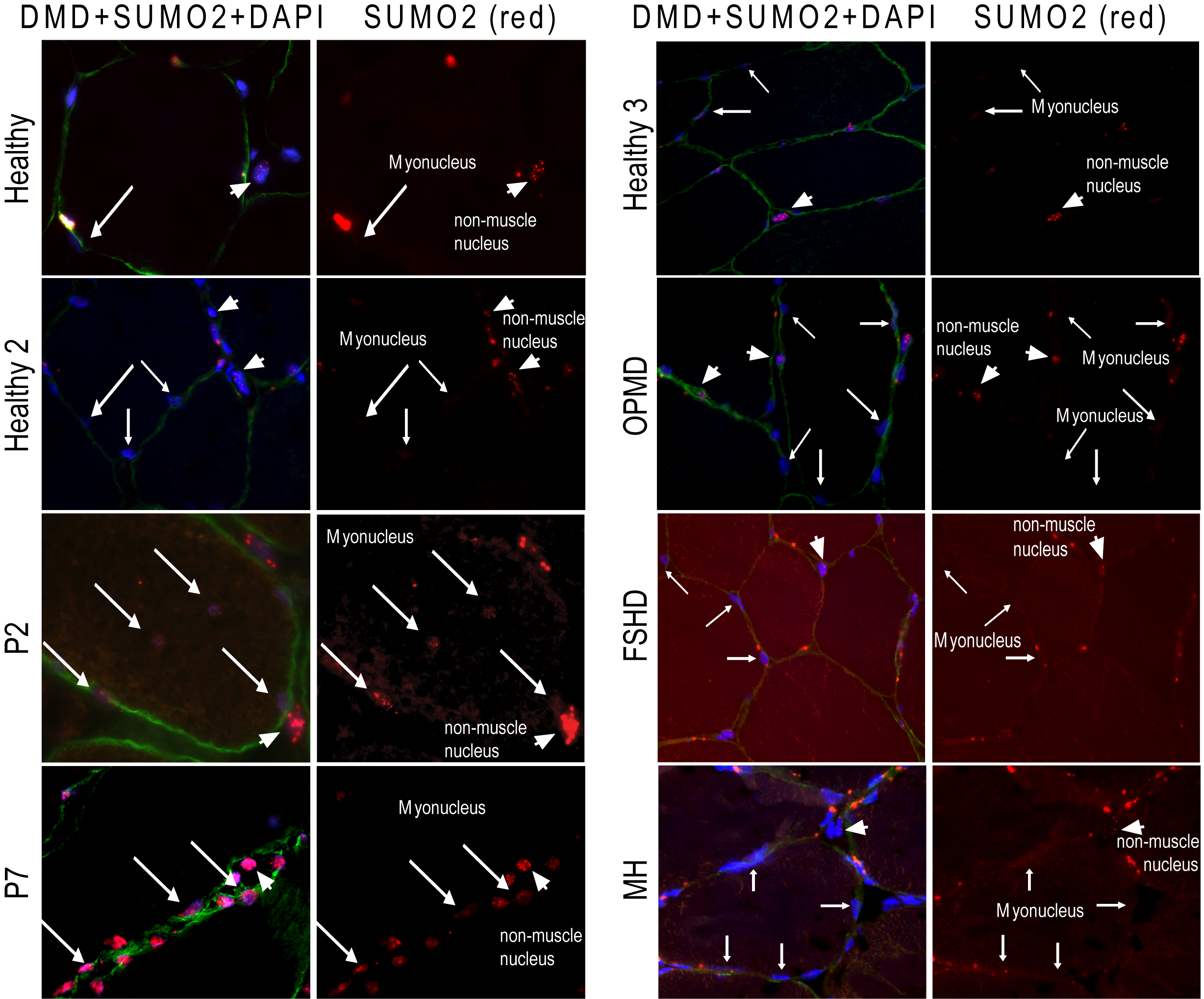

Supplement: Figure S5 — SUMO2 is increased in myonuclei of LGMD2A patients. Single cryosections of skeletal muscle of LGMD2a patients P2 and P7, three healthy controls, and three disease controls (MH, OPMD, FSHD) were stained for Dystrophin (DMD, green, muscle membrane marker) and SUMO2 (red). Nuclei are stained with DAPI in blue. Distinct nuclear dots are seen in non-muscle nuclei (Arrowheads), and in LGMD2A myonuclei (Large arrows). (4.59 MB TIF) [file pone.0011940.s005.tif]

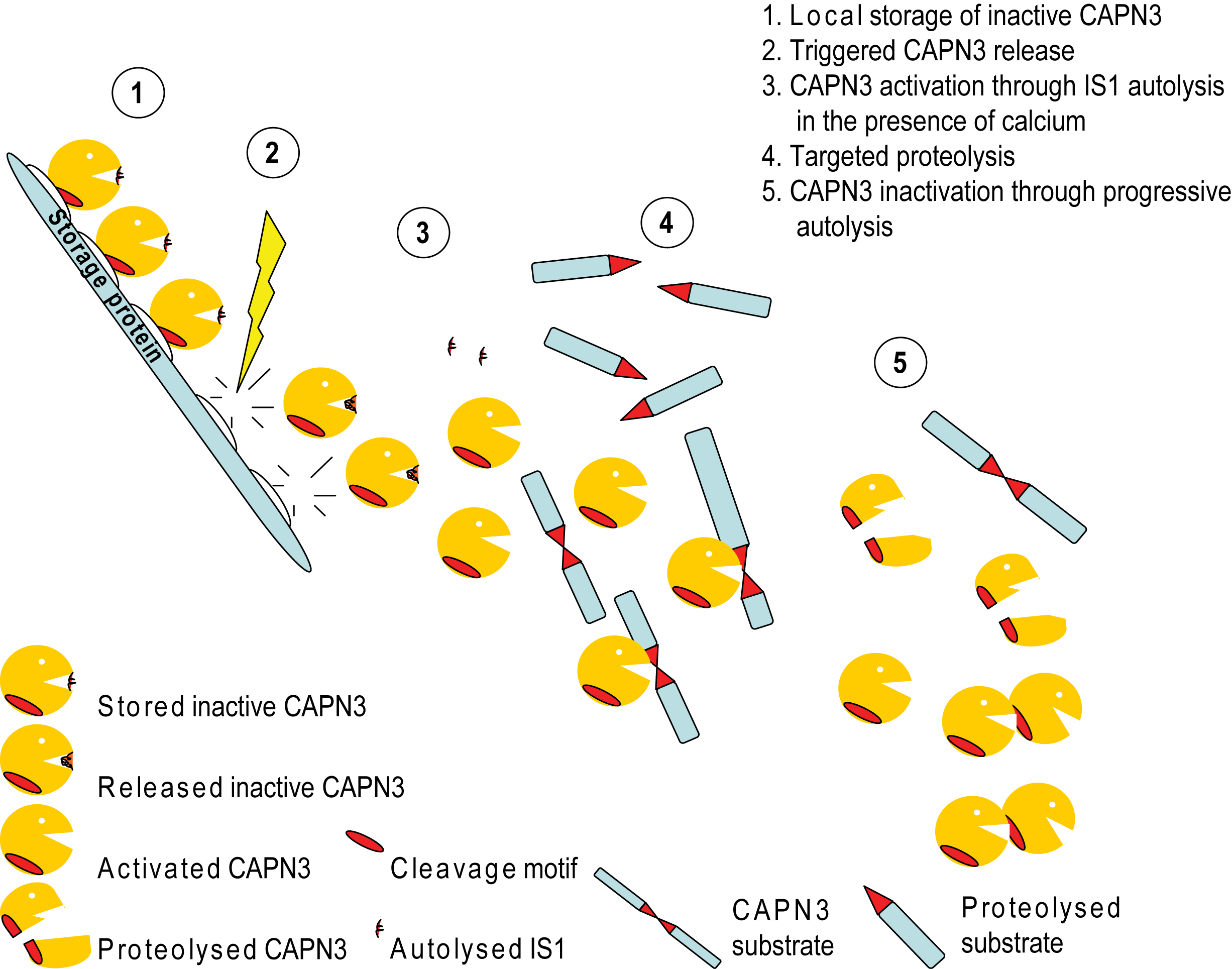

Supplement: Figure S6 — A model of CAPN3 function. Upon activation the chance that CAPN3 will encounter a substrate is enormous. The chance that the substrate is not CAPN3 is similarly high. However, as proteolysis proceeds and the number of non-processed substrates drops, the chance of CAPN3 encountering another CAPN3 increases. This will automatically control the number of active CAPN3 proteases inversely to the amount of processed substrates. Thus CAPN3 activity is local by default. (0.77 MB TIF) [file pone.0011940.s006.tif]
