# Supplementary material for: Calpain 3 Is a Rapid-Action, Unidirectional Proteolytic Switch Central to Muscle Remodeling
Source: PLoS One. 2010 Aug 4;5(8):e11940. doi: 10.1371/journal.pone.0011940 (PMC2915920; doi:10.1371/journal.pone.0011940)
Supplement: Table S1 — 325 predicted CAPN3 substrates. The specific motif sequence was screened against UniProt with the webtool ScanProsite, and yielded 325 unique proteins in human that contain the motif. For each of the 325 proteins the following information is listed: UniProt accession number, Protein ID, and Gene ID. For 18 of the proteins the crystal structure had been experimentally resolved. For these proteins a surface model of this structure in included below (hyperlink), with in red the cleavage motif. (1.72 MB DOC) [file pone.0011940.s007.doc]

| **Table S1: 325 predicted CAPN3 substrates** | | | |
| --- | --- | --- | --- |
| **Uniprot** | **ProtID** | **geneID** | **Crystal** |
| Q5TYW2 | A20A1 | **ANKRD20A1** |  |
| Q5SQ80 | A20A2 | **ANKRD20A2** |  |
| Q5VUR7 | A20A3 | **ANKRD20A3** |  |
| Q4UJ75 | A20A4 | **ANKRD20A4** |  |
| O94911 | **ABCA8** | **ABCA8** |  |
| Q8N961 | ABTB2 | **ABTB2** |  |
| Q15027 | ACAP1 | **ACAP1** |  |
| P36544 | ACHA7 | **CHRNA7** |  |
| Q8TDG2 | ACTT1 | **ACTRT1** |  |
| Q9BZ11 | ADA33 | **ADAM33** |  |
| Q99541 | ADFP | **ADFP** |  |
| Q8IUX7 | AEBP1 | **AEBP1** |  |
| Q8IVF2 | **AHNK2** | **AHNAK2** |  |
| Q09666 | **AHNK** | **AHNAK** |  |
| Q99996 | AKAP9 | **AKAP9** |  |
| Q53TS8 | AL2SA | **ALS2CR11** |  |
| Q9Y673 | ALG5 | **ALG5** |  |
| Q9H161 | ALX4 | **ALX4** |  |
| Q8IZ07 | AN13A | **ANKRD13A** |  |
| Q5CZ79 | AN20B | **ANKRD20B** |  |
| Q9UNK9 | **ANGE1** | **ANGEL1** |  |
| Q99873 | ANM1 | **PRMT1** | [1ORH.pdb](#OLE_LINK17) |
| Q9NR22 | ANM8 | **PRMT8** |  |
| Q9HCE9 | ANO8 | **ANO8** |  |
| Q10567 | **AP1B1** | **AP1B1** |  |
| P63010 | **AP2B1** | **AP2B1** | [1E42.pdb](#OLE_LINK3) |
| Q9NWB6 | ARGL1 | **ARGLU1** |  |
| Q8IUR7 | ARMC8 | **ARMC8** |  |
| Q9NQ33 | ASCL3 | **ASCL3** |  |
| Q9NR48 | ASH1L | **ASH1L** |  |
| Q14CW9 | AT7L3 | **ATXN7L3** |  |
| Q2TAZ0 | ATG2A | **ATG2A** |  |
| P82987 | ATL3 | **ADAMTSL3** |  |
| P25705 | ATPA | **ATP5A1** |  |
| P11274 | BCR | **BCR** |  |
| Q96CA5 | BIRC7 | **BIRC7** |  |
| Q9BWV1 | **BOC** | **BOC** |  |
| Q96RE7 | BTB14 | **BTBD14B** |  |
| Q9BZQ2 | CA014 | **C1orf14** |  |
| Q00975 | **CAC1B** | **CACNA1B** |  |
| P20807 | **CAN3** | **CAPN3** |  |
| A6NN90 | CB081 | **C2orf81** |  |
| Q92793 | CBP | **CREBBP** |  |
| Q9UK61 | CC063 | **C3orf63** |  |
| O60293 | CC131 | **CCDC131** |  |
| Q8IX12 | CCAR1 | **CCAR1** |  |
| Q5T9S5 | CCD18 | **CCDC18** |  |
| Q96HJ3 | CCD34 | **CCDC34** |  |
| A2IDD5 | CCD78 | **CCDC78** |  |
| Q86UT8 | CCD84 | **CCDC84** |  |
| Q8N998 | CCD89 | **CCDC89** |  |
| O15078 | CE290 | **CEP290** |  |
| Q96BT3 | CENPT | **CENPT** |  |
| Q53EZ4 | CEP55 | **CEP55** |  |
| Q5JTW2 | CEP78 | **CEP78** |  |
| Q9HD42 | CHM1A | **CHMP1A** |  |
| Q9H330 | CI005 | **C9orf5** |  |
| Q96MA6 | CI098 | **C9orf98** |  |
| Q9H8W3 | CJ084 | **C10orf84** |  |
| P35523 | CLCN1 | **CLCN1** |  |
| Q96LQ0 | CN050 | **C14orf50** |  |
| P42695 | CNDD3 | **NCAPD3** |  |
| Q6IBW4 | CNDH2 | **NCAPH2** |  |
| Q9H8Q6 | CO034 | **C15orf34** |  |
| Q7Z2Z1 | CO042 | **C15orf42** |  |
| Q01955 | CO4A3 | **COL4A3** |  |
| Q5TAT6 | CODA1 | **COL13A1** |  |
| P39059 | COFA1 | **COL15A1** |  |
| Q86Y22 | CONA1 | **COL23A1** |  |
| P53621 | COPA | **COPA** |  |
| Q9H0I2 | CP048 | **C16orf48** |  |
| Q9BV73 | CP250 | **CEP250** |  |
| Q9UBL6 | **CPNE7** | **CPNE7** |  |
| Q8IV36 | CQ028 | **C17orf28** |  |
| Q2NKJ3 | CQ068 | **C17orf68** |  |
| Q494W8 | CRFM7 | **CHRFAM7A** |  |
| Q5TZA2 | CROCC | **CROCC** |  |
| Q12996 | CSTF3 | **CSTF3** |  |
| P49711 | **CTCF** | **CTCF** |  |
| Q96P56 | CTSR2 | **CATSPER2** |  |
| Q9H467 | CUED2 | **CUEDC2** |  |
| Q9Y6V7 | DDX49 | **DDX49** |  |
| Q6ZUT9 | DEN5B | **DENND5B** |  |
| P23743 | DGKA | **DGKA** |  |
| Q9GZS0 | DNAI2 | **DNAI2** |  |
| Q8IZD9 | DOCK3 | **DOCK3** |  |
| Q99704 | DOK1 | **DOK1** |  |
| Q7Z5Q5 | DPOLN | **POLN** |  |
| Q16643 | DREB | **DBN1** |  |
| Q86SJ6 | DTWD2 | **DSG4** |  |
| Q0VDD8 | DYH14 | **DNAH14** |  |
| Q9C0G6 | DYH6 | **DNAH6** |  |
| Q9NR20 | DYRK4 | **DYRK4** |  |
| Q15075 | EEA1 | **EEA1** |  |
| Q9BSW2 | EFC4B | **EFCAB4B** |  |
| Q63HQ2 | EGFLA | **EGFLAM** |  |
| Q8NDI1 | EHBP1 | **EHBP1** |  |
| Q9NT22 | EMIL3 | **EMILIN3** |  |
| P42566 | EP15 | **EPS15** |  |
| Q09472 | EP300 | **EP300** |  |
| P58107 | EPIPL | **EPPK1** |  |
| P48449 | ERG7 | **LSS** |  |
| Q9NV70 | EXOC1 | **EXOC1** |  |
| Q8IXR5 | F178B | **FAM178B** |  |
| Q641Q2 | FA21A | **FAM21A** |  |
| Q5SNT6 | FA21B | **FAM21B** |  |
| Q9Y4E1 | FA21C | **FAM21C** |  |
| Q9NVI1 | FANCI | **FANCI** |  |
| Q6V0I7 | FAT4 | **FAT4** |  |
| Q8N3Y1 | FBXW8 | **FBXW8** |  |
| Q6BAA4 | FCRLB | **FCRLB** |  |
| O75369 | FLNB | **FLNB** | [2EEB.pdb](#OLE_LINK4) |
| Q14315 | **FLNC** | **FLNC** | [2D7P.pdb](#OLE_LINK18) |
| O95466 | FMNL | **FMNL1** |  |
| O94915 | **FRYL** | **FRYL** |  |
| Q5TBA9 | FRY | **FRY** |  |
| Q8N1E6 | FXL14 | **FBXL14** |  |
| Q9UN86 | G3BP2 | **G3BP2** |  |
| Q96RT7 | GCP6 | **TUBGCP6** |  |
| P43026 | GDF5 | **GDF5** |  |
| P10075 | GLI4 | **GLI4** |  |
| P36959 | GMPR1 | **GMPR** |  |
| Q9P2T1 | GMPR2 | **GMPR2** |  |
| Q13439 | GOGA4 | **GOLGA4** |  |
| Q96PE1 | GP124 | **GPR124** |  |
| Q6PRD1 | GP179 | **GPR179** |  |
| Q9Y3R0 | GRIP1 | **GRIP1** |  |
| Q9C0E4 | GRIP2 | **GRIP2** |  |
| Q9Y2T3 | GUAD | **GDA** |  |
| Q9ULT8 | HECD1 | **HECTD1** |  |
| O75031 | HSF2B | **HSF2BP** |  |
| Q7Z6Z7 | HUWE1 | **HUWE1** |  |
| P31269 | HXA9 | **HOXA9** |  |
| P20810 | **ICAL** | **CAST** |  |
| Q96RY7 | IF140 | **IFT140** |  |
| Q14164 | **IKKE** | **IKBKE** |  |
| Q9BZV3 | IMPG2 | **IMPG2** |  |
| Q9NVR2 | INT10 | **INTS10** |  |
| Q9Y2B9 | IPKG | **PKIG** |  |
| P17301 | ITA2 | **ITGA2** |  |
| P0C870 | JMJD7 | **JMJD7** |  |
| Q9BR39 | JPH2 | **JPH2** |  |
| Q7Z794 | K2C1B | **KRT77** |  |
| P08729 | K2C7 | **KRT7** |  |
| Q14678 | KANK1 | **KANK1** |  |
| Q63ZY3 | KANK2 | **KANK2** |  |
| Q5T7N3 | KANK4 | **KANK4** |  |
| Q9UJ90 | KCE1L | **KCNE1L** |  |
| O43525 | KCNQ3 | **KCNQ3** |  |
| Q7L273 | KCTD9 | **KCTD9** |  |
| Q2M1P5 | KIF7 | **KIF7** |  |
| Q5VTJ3 | KLD7A | **KLHDC7A** |  |
| A5PL33 | KRBA1 | **KRBA1** |  |
| P12956 | KU70 | **XRCC6** |  |
| P53671 | LIMK2 | **LIMK2** |  |
| Q5T7N2 | LITD1 | **L1TD1** |  |
| Q9BU23 | LMF2 | **LMF2** |  |
| O60711 | LPXN | **LPXN** |  |
| Q8N6Y2 | LRC17 | **LRRC17** |  |
| Q8IWT6 | LRC8A | **LRRC8A** |  |
| Q9C099 | LRCC1 | **LRRCC1** |  |
| O60341 | LSD1 | **AOF2** | Top of Form  [2H94.pdb](#OLE_LINK19) |
| Q99759 | M3K3 | **MAP3K3** |  |
| Q99683 | M3K5 | **MAP3K5** |  |
| Q8IWC1 | MA7D3 | **MAP7D3** |  |
| Q8WXG6 | MADD | **MADD** |  |
| Q16798 | MAON | **ME3** |  |
| P78559 | MAP1A | **MAP1A** |  |
| Q7KZI7 | MARK2 | **MARK2** |  |
| Q5U5Q3 | MEX3C | **MEX3C** |  |
| Q9UMN6 | MLL4 | **WBP7** |  |
| Q96T76 | MMS19 | **MMS19** |  |
| Q8NEH6 | MNS1 | **MNS1** |  |
| Q99550 | MPP9 | **MPHOSPH9** |  |
| Q96Q89 | MPPH1 | **MPHOSPH1** |  |
| Q5VT25 | **MRCKA** | **CDC42BPA** |  |
| Q9Y5S2 | MRCKB | **CDC42BPB** |  |
| Q6DT37 | MRCKG | **CDC42BPG** |  |
| Q7Z406 | MYH14 | **MYH14** |  |
| P32418 | **NAC1** | **SLC8A1** |  |
| Q13772 | NCOA4 | **NCOA4** |  |
| O75376 | NCOR1 | **NCOR1** |  |
| Q9Y618 | NCOR2 | **NCOR2** |  |
| Q9ULJ8 | NEB1 | **PPP1R9A** |  |
| Q8NG66 | NEK11 | **NEK11** |  |
| P48681 | NEST | **NES** |  |
| P07196 | NFL | **NEFL** |  |
| Q86UT6 | NLRX1 | **NLRX1** |  |
| Q13253 | NOGG | **NOG** | [1M4U.pdb](#OLE_LINK5) |
| Q9Y2X3 | NOL5 | **NOL5** |  |
| Q92621 | NU205 | **NUP205** |  |
| Q14980 | NUMA1 | **NUMA1** |  |
| Q9ULJ1 | ODF2L | **ODF2L** |  |
| Q9BXW6 | OSBL1 | **OSBPL1A** |  |
| O14832 | PAHX | **PHYH** | [2A1X.pdb](#OLE_LINK6) |
| Q53GL7 | PAR10 | **PARP10** |  |
| Q9UKK3 | PARP4 | **PARP4** |  |
| Q96RG2 | PASK | **PASK** |  |
| P15863 | PAX1 | **PAX1** |  |
| P49023 | PAXI | **PXN** |  |
| P29122 | PCSK6 | **PCSK6** |  |
| P16499 | PDE6A | **PDE6A** |  |
| O60437 | PEPL | **PPL** |  |
| P00558 | PGK1 | **PGK1** | [3C39.pdb](#OLE_LINK7) |
| P07205 | PGK2 | **PGK2** | [2P9Q.pdb](#OLE_LINK8) |
| Q9Y6X2 | **PIAS3** | **PIAS3** |  |
| Q9ULM0 | PKHH1 | **PLEKHH1** |  |
| Q8IWE5 | PKHM2 | **PLEKHM2** |  |
| Q8N7P1 | PLD5 | **PLD5** |  |
| Q15149 | PLEC1 | **PLEC1** | [2ODU.pdb](#OLE_LINK9) |
| P13796 | PLSL | **LCP1** |  |
| P13797 | PLST | **PLS3** |  |
| Q8WZA1 | **PMGT1** | **POMGNT1** |  |
| Q96KW2 | PO122 | **POM121L2** |  |
| Q4J6C6 | PPCEL | **PREPL** |  |
| Q9UNP9 | PPIE | **PPIE** | [2CQB.pdb](#OLE_LINK10) |
| Q5SGD2 | **PPM1L** | **PPM1L** |  |
| O95522 | PRA12 | **PRAMEF12** |  |
| A6NMC2 | PRA24 | **PRAMEF24** |  |
| Q5VXH5 | PRAM7 | **PRAMEF7** |  |
| Q5VWM4 | PRAM8 | **PRAMEF8** |  |
| Q9BXM0 | PRAX | **PRX** |  |
| Q9UBK2 | PRGC1 | **PPARGC1A** |  |
| P61289 | PSME3 | **PSME3** |  |
| Q8N8N7 | PTGR2 | **PTGR2** | [2ZB8.pdb](#OLE_LINK11) |
| Q12923 | PTN13 | **PTPN13** |  |
| P11216 | PYGB | **PYGB** |  |
| P06737 | PYGL | **PYGL** |  |
| P11217 | **PYGM** | **PYGM** |  |
| Q5TB80 | QN1 | **KIAA1009** |  |
| Q96JH8 | RADIL | **RADIL** | [3EC8.pdb](#OLE_LINK12) |
| Q15283 | RASA2 | **RASA2** |  |
| P50749 | RASF2 | **RASSF2** |  |
| Q8TDY2 | RBCC1 | **RB1CC1** |  |
| Q7Z3Z2 | RD3 | **RD3** |  |
| P82980 | RET5 | **RBP5** |  |
| Q86YS3 | RFIP4 | **RAB11FIP4** |  |
| Q5TG30 | RG18L | **C20orf95** |  |
| Q13017 | RHG05 | **ARHGAP5** |  |
| Q52LW3 | RHG29 | **ARHGAP29** |  |
| Q13671 | RIN1 | **RIN1** |  |
| Q13464 | ROCK1 | **ROCK1** |  |
| O75116 | ROCK2 | **ROCK2** |  |
| Q8IWN7 | RP1L1 | **RP1L1** |  |
| Q9Y4G8 | RPGF2 | **RAPGEF2** |  |
| Q9P2E9 | RRBP1 | **RRBP1** |  |
| Q96T23 | RSF1 | **RSF1** |  |
| P23297 | S10A1 | **S100A1** | [1K2H.pdb](#OLE_LINK13) |
| O15244 | S22A2 | **SLC22A2** |  |
| Q695T7 | S6A19 | **SLC6A19** |  |
| Q9NP91 | S6A20 | **SLC6A20** |  |
| Q9NZJ4 | SACS | **SACS** |  |
| P07602 | SAP | **PSAP** |  |
| Q6UVJ0 | SAS6 | **SASS6** |  |
| Q12770 | SCAP | **SCAP** |  |
| P13521 | SCG2 | **SCG2** |  |
| Q9NQ36 | SCUB2 | **SCUBE2** |  |
| Q6P3W7 | SCYL2 | **SCYL2** |  |
| Q13275 | SEM3F | **SEMA3F** |  |
| Q9NVA2 | SEP11 | **SEPT11** |  |
| Q12884 | SEPR | **FAP** |  |
| Q14141 | SEP06 | **SEPT6** |  |
| Q92599 | SEP08 | **SEPT8** |  |
| O15357 | SHIP2 | **INPPL1** |  |
| Q6PI26 | SHQ1 | **SHQ1** |  |
| Q9H2G2 | SLK | **SLK** |  |
| Q9NZC9 | SMAL1 | **SMARCAL1** |  |
| Q3KNW1 | SNAI3 | **SNAI3** |  |
| Q9Y5W7 | SNX14 | **SNX14** |  |
| P57768 | SNX16 | **SNX16** |  |
| Q7KZ85 | SPT6H | **SUPT6H** |  |
| Q13813 | **SPTA2** | **SPTAN1** | [2JM9.pdb](#OLE_LINK14) |
| P11277 | SPTB1 | **SPTB** |  |
| Q96JI7 | SPTCS | **SPG11** |  |
| Q9NRC6 | SPTN5 | **SPTBN5** |  |
| Q9UHB9 | SRP68 | **SRP68** |  |
| P28290 | SSFA2 | **SSFA2** |  |
| Q92502 | STAR8 | **STARD8** |  |
| O94804 | STK10 | **STK10** |  |
| Q7Z7C7 | STRA8 | **STRA8** |  |
| Q9P2W9 | STX18 | **STX18** |  |
| P26640 | SYVC | **VARS** |  |
| Q969Z0 | TBRG4 | **TBRG4** |  |
| O14559 | TCGAP | **SNX26** |  |
| Q9BT92 | TCHP | **TCHP** |  |
| O14746 | TERT | **TERT** |  |
| O43151 | TET3 | **TET3** |  |
| Q12789 | TF3C1 | **GTF3C1** |  |
| Q9UK28 | TM59L | **TMEM59L** |  |
| Q9HD45 | TM9S3 | **TM9SF3** |  |
| A8MYB1 | TMC5B | **TMCO5B** |  |
| A0PK05 | TMM72 | **TMEM72** |  |
| Q9H2K2 | TNKS2 | **TNKS2** |  |
| P02585 | TNNC2 | **TNNC2** | [1YV0.pdb](#OLE_LINK15) |
| P07951 | **TPM2** | **TPM2** |  |
| P12270 | TPR | **TPR** |  |
| Q96PN7 | TREF1 | **TRERF1** |  |
| Q9C019 | TRI15 | **TRIM15** |  |
| O94972 | TRI37 | **TRIM37** |  |
| Q6ZTA4 | TRI67 | **TRIM67** |  |
| A6NK02 | TRI75 | **TRIM75** |  |
| Q9HBA0 | TRPV4 | **TRPV4** |  |
| Q9NQA5 | TRPV5 | **TRPV5** |  |
| Q9H1D0 | TRPV6 | **TRPV6** |  |
| Q2NL82 | TSR1 | **TSR1** |  |
| Q8NDW8 | TT21A | **TTC21A** |  |
| O95551 | TTRAP | **TTRAP** |  |
| Q9H6E5 | TUT1 | **TUT1** |  |
| O14530 | TXND9 | **TXNDC9** |  |
| Q86V40 | U632B | **UPF0632 protein B** |  |
| Q5JPF3 | U634C | **UPF0634 protein C** |  |
| Q5T4S7 | UBR4 | **UBR4** |  |
| P46939 | UTRO | **UTRN** |  |
| O95498 | VNN2 | **VNN2** |  |
| Q6ZS81 | WDFY4 | **WDFY4** |  |
| Q562E7 | WDR81 | **WDR81** |  |
| Q8IX03 | WWC1 | **WWC1** |  |
| Q13426 | **XRCC4** | **XRCC4** | [1FU1.pdb](#OLE_LINK16) |
| Q6ZMT9 | YD026 | **FLJ16686** |  |
| A6NC05 | YD286 | **YDR286C** |  |
| P37275 | ZEB1 | **ZEB1** |  |
| P52739 | ZN131 | **ZNF131** |  |
| Q96JG9 | ZN469 | **ZNF469** |  |
| Q9Y462 | ZN711 | **ZNF711** |  |
| O43149 | ZZEF1 | **ZZEF1** |  |


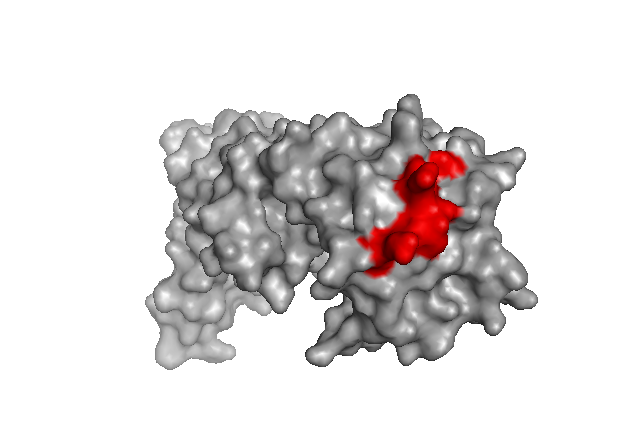


ANM1

1ORH.pdb


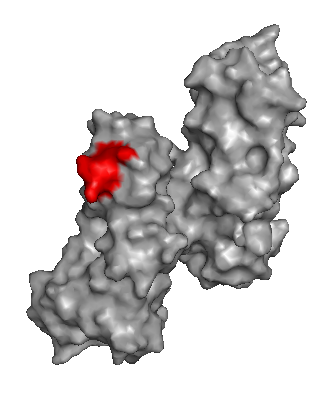


**AP2B1**

[1E42.pdb](#OLE_LINK3)


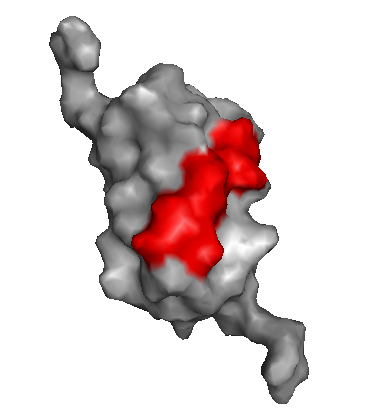


**FLNB**

2EEB.pdb


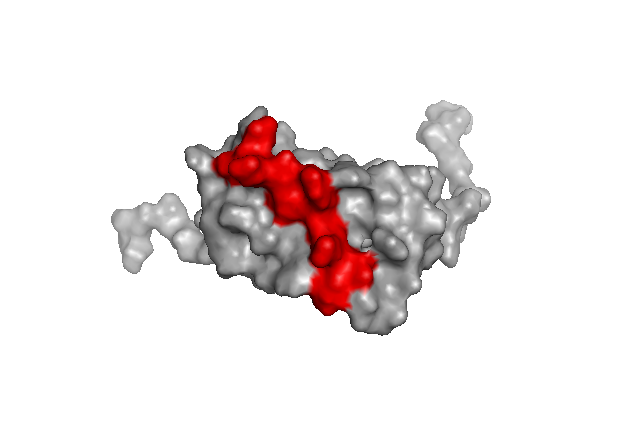


**FLNC**

2D7P.pdb


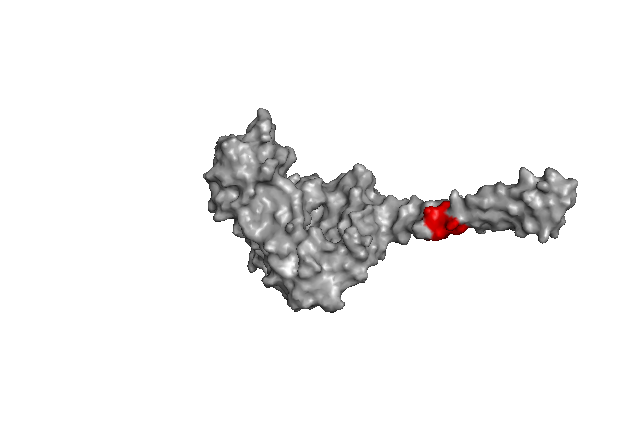


LSD1

2H94.pdb


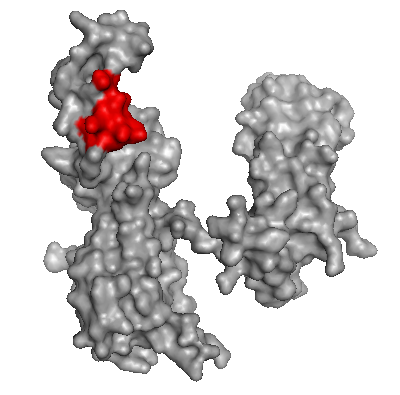


NOGG

1M4U.pdb


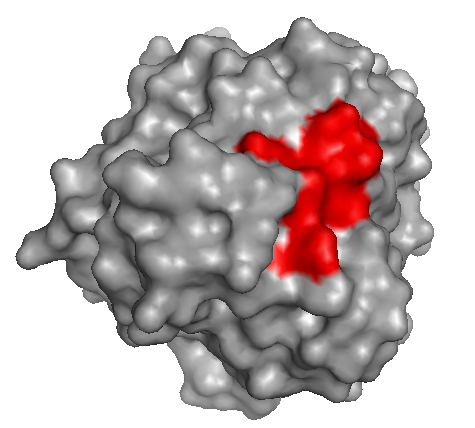


PAHX

2A1X.pdb


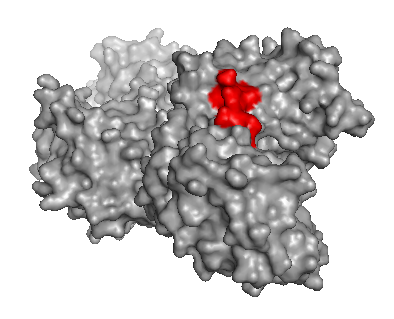


PGK1

3C39.pdb


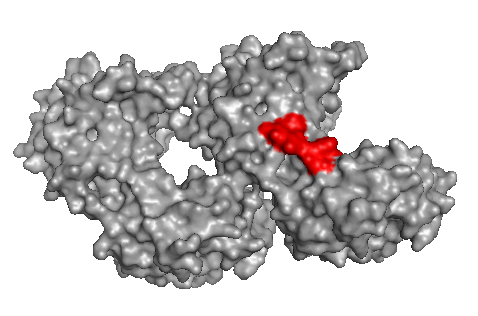


PGK2

2P9Q.pdb


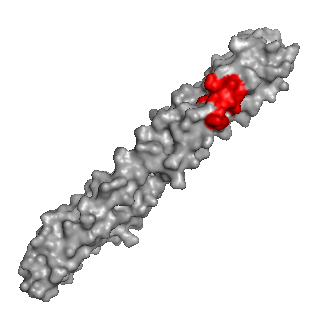


PLEC1

2ODU.pdb


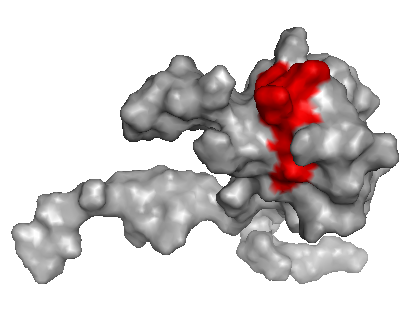


PPIE

2CQB.pdb


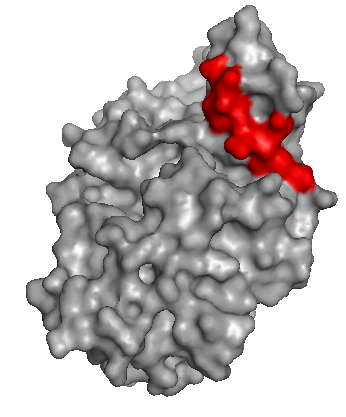


PTGR2

2ZB8.pdb


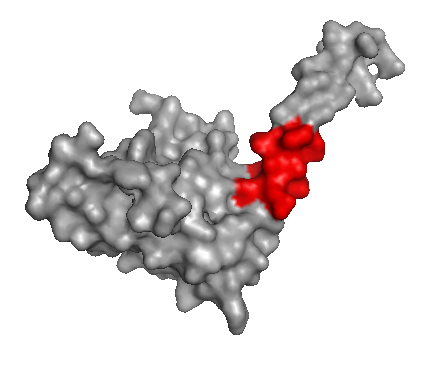


RADIL

3EC8.pdb


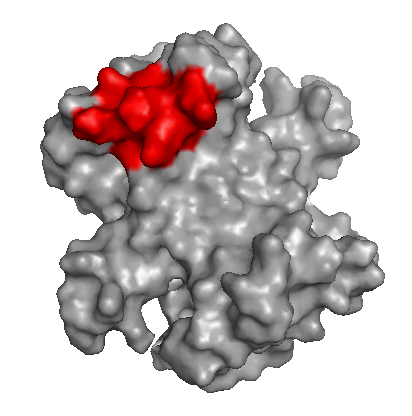


S10A1

1K2H.pdb


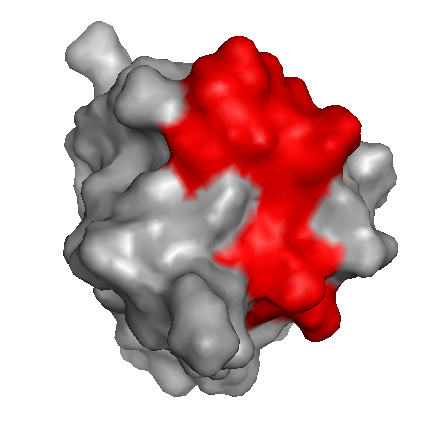


**SPTA2**

2JM9.pdb


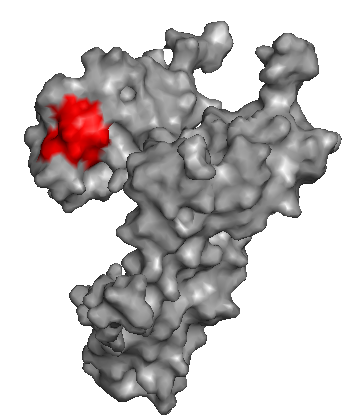


TNNC2

1YV0.pdb


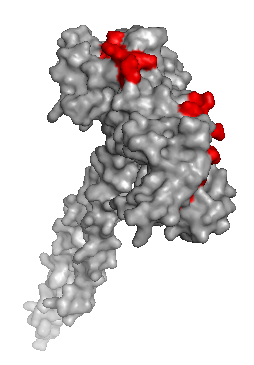


**XRCC4**

1FU1.pdb
