# Supplementary material for: Calpain 3 Is a Rapid-Action, Unidirectional Proteolytic Switch Central to Muscle Remodeling
Source: PLoS One. 2010 Aug 4;5(8):e11940. doi: 10.1371/journal.pone.0011940 (PMC2915920; doi:10.1371/journal.pone.0011940)
Supplement: Table S2 — Conceptual clusters occurring in the list of putative CAPN3 substrates. The 325 putative substrates were loaded into the software program Anni and associated with concepts. The concepts associated with the 325 putative substrates were grouped into clusters and statistically weighed. The most significantly occurring clusters were annotated with concepts. The first column list the statistically weighed clusters, with in the second column the associated concepts. The third column shows the genes belonging to each cluster; with in bold those that tested positive in the fusion protein assay. (0.29 MB DOC) [file pone.0011940.s008.doc]

| **Table S2: Conceptual clusters occurring in the list of putative CAPN3 substrates** | | |
| --- | --- | --- |
| **Clusters** | **Associated Concepts** | **Genes** |
| **Mitotic spindle** | Centrosome | NUMA1 |
|  | Centrioles | CROCC |
|  | midbody | CEP250 |
|  | ESCRT III complex location | SASS6 |
|  | spindle pole | AKAP9 |
|  | centrosome duplication | CEP55 |
|  | ciliary rootlet | PCOLN3 |
|  | Mitotic Spindle Apparatus | FMNL1 |
|  | pericentriolar material |  |
|  | HIV Budding |  |
|  | centriole replication |  |
|  | centrosome separation |  |
|  | Mitotic spindle |  |
| **Malignant neoplasms** | bcr-abl peptide vaccine | FLNB |
|  | Myeloid Leukemia, Chronic | GOLGA4 |
|  | small nucleolar ribonucleoprotein activity | BCR |
|  | Small Nucleolar Ribonucleoproteins | NOP5/NOP58 |
|  | small nucleolar ribonucleoprotein complex location | DOK1 |
|  | imatinib | TPR |
|  | DNA Sequence Rearrangement | Ncoa4 |
|  | Philadelphia Chromosome |  |
|  | Tyrosine |  |
|  | Fusion Protein |  |
|  | Gene Rearrangement |  |
|  | Oncogene Proteins, Fusion |  |
|  | Oncogene Proteins |  |
|  | Autoantigens |  |
|  | Oncogenes |  |
|  | Proto-Oncogene Proteins |  |
|  | Thyroid Gland |  |
| **Vesicular protein transport** | Clathrin | EXOC7 |
|  | Endosomes | GDA |
|  | Vesicular Protein Transport | CENTB1 |
|  | Vesicular Transport Proteins | EPS15 |
|  | exocyst | **AP2B1** |
|  | Adaptor Signaling Protein | EEA1 |
|  | Endocytosis | **AP1B1** |
|  | Adaptor Protein Complex 1 | STX18 |
|  | Adaptor Protein Complex alpha Subunits |  |
|  | adaptin location |  |
|  | ADP-Ribosylation Factors |  |
|  | Clathrin-Coated Vesicles |  |
|  | clathrin vesicle coat |  |
|  | Membrane Protein Traffic |  |
|  | clathrin coat |  |
|  | Recycling |  |
|  | Adaptor Proteins, Vesicular Transport |  |
|  | Early endosome |  |
|  | coated pit |  |
| **Phosphatidylinositol phosphate** | phosphatidylinositol 3,4,5-triphosphate | RAB11FIP4 |
|  | inositol-1,3,4,5-tetrakisphosphate | INPPL1 |
|  | phosphatidylinositol 3,4-diphosphate | RASA2 |
|  | Phosphoric Monoester Hydrolases | Copa |
|  | phosphoinositide binding | PLEKHM2 |
|  | membrane ruffle |  |
|  | phosphatidylinositol phosphate, PtdIns(4,5)P2 |  |
| **Intermediate filament organisation** | Plakins | S100A1 |
|  | Plectin | Mtap1a |
|  | Intermediate Filament Proteins | NEFL |
|  | Desmogleins | NES |
|  | Intermediate Filaments | KRT7 |
|  | Keratin | DSG4 |
|  | Neurofilament | **PPL** |
|  | Desmosomes | EPPK1 |
|  | S100 Proteins | PLEC1 |
|  | glial fibrillary acidic protein location | **AHNAK** |
|  | Neurofilament-L | PRX |
|  | Microtubule-Associated Proteins |  |
|  | Cytoskeletal Proteins |  |
| **Actin cytoskeleton** | Muscular Dystrophies, Limb-Girdle | **SPTAN1** |
|  | actin binding | SPTB |
|  | Intracellular Signaling Peptides and Proteins | **CAST** |
|  | Muscular Dystrophies | **FLNC** |
|  | Sarcoglycans | UTRN |
|  | Thrombin | **CAPN3** |
|  | Stress Fibers | PPP1R9A |
|  | F-Actin | DBN1 |
|  | Troponin T | LCP1 |
|  | Protein-Serine-Threonine Kinases | PLS3 |
|  | Protein Tyrosine Phosphatase | **TPM2** |
|  |  | TNNC2 |
|  |  | MARK2 |
|  |  | ITGA2 |
|  |  | PXN |
|  |  | ROCK1 |
|  |  | ROCK2 |
|  |  | LPXN |
|  |  | LIMK2 |
|  |  | PTPN13 |
| **Apoptosis** | MAP Kinase Kinase Kinase | MAP3K5 |
|  | inhibitor-of-apoptosis protein | MAP3K3 |
|  | polo-like kinase kinase 1 | PSME3 |
|  | proteasome activator activity | STK10 |
|  | proteasome activator complex location | SLK |
|  | Adaptor Proteins, Signal Transducing | MADD |
|  | MAP Kinase Cascade | BIRC7 |
|  | MP kinase activity |  |
|  | SAPK |  |
|  | Death Domain |  |
|  | Mitogen-Activated Protein Kinases |  |
|  | caspase |  |
|  | Intracisternal A-Particle Elements |  |
|  | upstream kinase |  |
| **DNA binding** | Tankyrases | TERT |
|  | Nonhomologous DNA End Joining | XRCC6 |
|  | telomere | **XRCC4** |
|  | Poly Adenosine Diphosphate Ribose | PARP10 |
|  | DNA-dependent protein kinase | TNKS2 |
|  | DNA Breaks, Double-Stranded | PARP4 |
|  | Vault |  |
|  | DNA-Binding Proteins |  |
|  | telomerase activity |  |
|  | Polymerase |  |
|  | DNA Repair |  |
|  | Genomic Stability |  |
|  | V(D)J Recombination |  |
|  | Telomere Maintenance |  |
|  | Telomere Length Maintenance |  |
| **Transcription regulation** | Histone Deacetylase | ME3 |
|  | Trans-Activation (Genetics) | AOF2 |
|  | Bone Morphogenetic Proteins | ASH1L |
|  | Receptors, Nuclear | MLL4 |
|  | Histone H3 | NCOR1 |
|  | transcription factor | EP300 |
|  | PHD Finger | CREBBP |
|  | Repressor Proteins | NCOR2 |
|  | Transcriptional Repression | CTCF |
|  | nuclear receptor co-repressor | GRIP1 |
|  | Protein Inhibitors of Activated STAT | PRMT1 |
|  | Cyclic AMP-Responsive DNA-Binding Protein | PPARGC1A |
|  | Repression | PPIE |
|  | Protein-Arginine N-Methyltransferase | RSF1 |
|  | Acetylation | **PIAS3** |
|  | Oncogene E1A | RB1CC1 |
|  | protein sumoylation | NOG |
|  | Nuclear Proteins | Gdf5 |
|  | polycomb group proteins | PAX1 |
|  | Histones |  |
|  | chromatin immunoprecipitation |  |
|  | histone methyltransferase |  |
|  | Transcription, Genetic |  |
|  | histone modification |  |
|  | chromatin remodeling |  |
|  | Thyroid Hormone Receptor |  |
| **Lipid metabolism** | Sterols | GRIP2 |
|  | Lipid droplet | LSS |
|  | Mutase | SCAP |
|  | cholesterol biosynthesis | ADFP |
|  | Sterol Regulatory Element Binding Proteins | Osbpl1a |
|  | 25-hydroxycholesterol |  |
|  | Intracellular Signaling Peptides and Proteins |  |
|  | liver X receptor |  |
|  | Lipogenesis |  |
|  | Lanosterol |  |
|  | cellular lipid metabolism |  |
|  | Ligand Binding Domain |  |
|  | Foam Cells |  |
|  | Lipid Metabolism |  |
| **Serine protease activity** | Proprotein Convertases | TSR1 |
|  | ADAMTS | ADAM33 |
|  | ADAM Proteins | SCG2 |
|  | serine endopeptidase | PCSK6 |
|  | Disintegrins | SSFA2 |
|  | Subtilisins | FAP |
|  | Subtilisin |  |
|  | endopeptidase activity |  |
|  | Dipeptidyl Peptidases |  |
|  | Prohormone Convertases |  |
|  | Chromogranins |  |
|  | Metalloproteases |  |
|  | Serine Protease |  |
|  | Activin Receptor |  |
| **Ion transport** | Calcium Channel | TRPV5 |
|  | TRP Channel | TRPV4 |
|  | infliximab | TRPV6 |
|  | voltage gated channel | **CACNA1B** |
|  | calbindin | SLC8A1 |
|  | Patch-Clamp Techniques | CATSPER2 |
|  | Ion Channel Gating | KCNE1L |
|  | Gated Ion Channel | KCNQ3 |
|  | Vanilloid | CLCN1 |
|  | Ion Channel |  |
|  | Cations |  |
|  | Transient Receptor Potential Channels |  |
|  | KCNQ1 Potassium Channel |  |
|  | calcium:sodium antiporter activity |  |
|  | Amino Acid Transport Systems, Basic |  |
|  | Chloride Channels |  |
|  | exchanger |  |
|  | Channelopathies |  |
|  | Calcium |  |
| **Amino acid transport** | Amino Acid Transport Systems, Neutral | INTS10 |
|  | 2p21 | PREPL |
|  | cystinuria | Slc6a20 |
|  | neutral amino acid transport | SLC6A19 |
|  | amino acid transport |  |
|  | Neurotransmitter Transport Proteins |  |
|  | Imino Acids |  |
|  | Amino Acids, Neutral |  |
| **Ocular physiology** | Leber's disease | IMPG2 |
|  | Retinal Degeneration | CEP290 |
|  | Photoreceptors, Vertebrate | PDE6A |
|  | Retinal Dystrophy | rd3 |
|  | Retinitis Pigmentosa |  |
|  | Rod Photoreceptors |  |
|  | Mycobacterium tuberculosis complex |  |
|  | Photoreceptors |  |
|  | Eye Proteins |  |
|  | 1q41 |  |
|  | photoreceptor outer segment |  |
|  | Structure of outer nuclear layer of retina |  |
|  | visual photoreceptor |  |
