# Supplementary material for: Calpain 3 Is a Rapid-Action, Unidirectional Proteolytic Switch Central to Muscle Remodeling
Source: PLoS One. 2010 Aug 4;5(8):e11940. doi: 10.1371/journal.pone.0011940 (PMC2915920; doi:10.1371/journal.pone.0011940)
Supplement: Table S3 — GO term annotation analysis for the putative CAPN3 substrates. Gene Ontology pathway analysis was performed on the set of 325 putative substrates, to identity those pathways that are most informative for the set. The first column shows the entropy, where high entropy means a high content of information. The second column lists the calculated numbers (Substrates belonging to GO term | Absolute number of proteins belonging to GO term pathway | Total proteins associated with GO term | Total substrates in GO term pathway). In addition, the corresponding GO term ID, GO Pathway, and GO term are given in the last three columns. The top 20 hits for the Pathway Cellular Component are plotted in Figure S2c. The Table was cut at an entropy of 2.2669. (0.13 MB DOC) [file pone.0011940.s009.doc]

**Table S3**

| Entropy | Substrates belonging to GO term | Absolute number of proteins belonging to GO term pathway | Total proteins associated with GO term | Total substrates in GO term pathway | GO ID | Pathway | GO term |
| --- | --- | --- | --- | --- |

26.69 49|15182|1046|239 GO:0005856 cellular_component cytoskeleton

22.74 158|14596|6753|238 GO:0005515 molecular_function protein binding

20.58 59|15182|1680|239 GO:0043228 cellular_component non-membrane-bounded organelle

20.58 59|15182|1680|239 GO:0043232 cellular_component intracellular non-membrane-bounded organelle

19.12 33|15182|675|239 GO:0044430 cellular_component cytoskeletal part

19.04 45|13581|1127|218 GO:0006996 biological_process organelle organization and biogenesis

15.30 217|14596|11545|238 GO:0005488 molecular_function binding

15.17 190|15182|9849|239 GO:0005622 cellular_component intracellular

15.15 2|14596|1201|238 GO:0004888 molecular_function transmembrane receptor activity

14.36 8|15182|47|239 GO:0044448 cellular_component cell cortex part

14.06 22|14596|411|238 GO:0008092 molecular_function cytoskeletal protein binding

12.73 23|13581|491|218 GO:0007010 biological_process cytoskeleton organization and biogenesis

12.63 17|14596|284|238 GO:0003779 molecular_function actin binding

12.59 52|13581|1752|218 GO:0016043 biological_process cellular component organization and biogenesis

12.36 3|14596|3|238 GO:0008184 molecular_function glycogen phosphorylase activity

12.19 180|15182|9450|239 GO:0044424 cellular_component intracellular part

11.88 2|13581|1007|218 GO:0007186 biological_process G-protein coupled receptor protein signaling pathway

11.83 0|14596|702|238 GO:0001584 molecular_function rhodopsin-like receptor activity

11.64 8|15182|67|239 GO:0005938 cellular_component cell cortex

11.05 1|14596|810|238 GO:0004930 molecular_function G-protein coupled receptor activity

10.71 13|13581|206|218 GO:0030036 biological_process actin cytoskeleton organization and biogenesis

10.64 9|14596|1629|238 GO:0004872 molecular_function receptor activity

10.45 28|13581|774|218 GO:0007049 biological_process cell cycle

10.09 3|14596|5|238 GO:0004645 molecular_function phosphorylase activity

9.98 7|14596|60|238 GO:0019992 molecular_function diacylglycerol binding

9.82 13|13581|225|218 GO:0030029 biological_process actin filament-based process

9.78 46|15182|1673|239 GO:0043234 cellular_component protein complex

9.71 12|13581|196|218 GO:0016568 biological_process chromatin modification

9.59 125|15182|6179|239 GO:0005737 cellular_component cytoplasm

9.54 31|13581|951|218 GO:0043687 biological_process post-translational protein modification

9.33 17|15182|384|239 GO:0015630 cellular_component microtubule cytoskeleton

9.09 18|14596|416|238 GO:0042802 molecular_function identical protein binding

9.09 10|15182|151|239 GO:0005815 cellular_component microtubule organizing center

8.80 54|15182|4966|239 GO:0031224 cellular_component intrinsic to membrane

8.70 16|14596|2048|238 GO:0060089 molecular_function molecular transducer activity

8.70 16|14596|2048|238 GO:0004871 molecular_function signal transducer activity

8.57 53|15182|4865|239 GO:0016021 cellular_component integral to membrane

8.52 16|13581|367|218 GO:0051276 biological_process chromosome organization and biogenesis

8.46 3|13581|8|218 GO:0031532 biological_process actin cytoskeleton reorganization

8.44 14|15182|1929|239 GO:0005576 cellular_component extracellular region

8.36 230|15182|13695|239 GO:0044464 cellular_component cell part

8.35 230|15182|13696|239 GO:0005623 cellular_component cell

8.24 2|14596|2|238 GO:0016657 molecular_function "oxidoreductase activity, acting on NADH or NADPH, nitrogenous group as acceptor"

8.24 2|14596|2|238 GO:0003920 molecular_function GMP reductase activity

8.24 2|14596|2|238 GO:0004618 molecular_function phosphoglycerate kinase activity

8.16 9|15182|139|239 GO:0005813 cellular_component centrosome

8.13 3|15182|9|239 GO:0008091 cellular_component spectrin

7.87 52|15182|2149|239 GO:0032991 cellular_component macromolecular complex

7.86 5|13581|40|218 GO:0016570 biological_process histone modification

7.64 5|13581|42|218 GO:0016569 biological_process covalent chromatin modification

7.49 3|15182|11|239 GO:0031105 cellular_component septin complex

7.49 3|15182|11|239 GO:0032156 cellular_component septin cytoskeleton

7.43 3|13581|11|218 GO:0016573 biological_process histone acetylation

7.19 2|13581|3|218 GO:0018076 biological_process N-terminal peptidyl-lysine acetylation

7.19 2|13581|3|218 GO:0018394 biological_process peptidyl-lysine acetylation

7.18 16|14596|411|238 GO:0004674 molecular_function protein serine/threonine kinase activity

7.14 35|14596|1279|238 GO:0005524 molecular_function ATP binding

7.03 24|13581|778|218 GO:0016310 biological_process phosphorylation

6.96 35|13581|1318|218 GO:0006464 biological_process protein modification process

6.93 36|13581|1371|218 GO:0043412 biological_process biopolymer modification

6.93 22|14596|680|238 GO:0005198 molecular_function structural molecule activity

6.92 35|14596|1297|238 GO:0032559 molecular_function adenyl ribonucleotide binding

6.92 21|13581|647|218 GO:0006468 biological_process protein amino acid phosphorylation

6.88 27|13581|929|218 GO:0006796 biological_process phosphate metabolic process

6.88 27|13581|929|218 GO:0006793 biological_process phosphorus metabolic process

6.87 13|14596|305|238 GO:0008047 molecular_function enzyme activator activity

6.79 6|15182|77|239 GO:0005929 cellular_component cilium

6.55 10|15182|212|239 GO:0015629 cellular_component actin cytoskeleton

6.55 11|13581|245|218 GO:0045893 biological_process "positive regulation of transcription, DNA-dependent"

6.49 11|13581|247|218 GO:0051254 biological_process positive regulation of RNA metabolic process

6.48 12|14596|281|238 GO:0003712 molecular_function transcription cofactor activity

6.48 2|14596|4|238 GO:0016774 molecular_function "phosphotransferase activity, carboxyl group as acceptor"

6.47 14|14596|360|238 GO:0008289 molecular_function lipid binding

6.46 26|14596|895|238 GO:0016772 molecular_function "transferase activity, transferring phosphorus-containing groups"

6.38 14|14596|364|238 GO:0030695 molecular_function GTPase regulator activity

6.34 3|15182|16|239 GO:0030864 cellular_component cortical actin cytoskeleton

6.34 4|14596|33|238 GO:0035258 molecular_function steroid hormone receptor binding

6.29 3|13581|16|218 GO:0007076 biological_process mitotic chromosome condensation

6.26 7|13581|114|218 GO:0006816 biological_process calcium ion transport

6.26 12|13581|294|218 GO:0006325 biological_process establishment or maintenance of chromatin architecture

6.22 40|14596|1616|238 GO:0016740 molecular_function transferase activity

6.22 0|13581|379|218 GO:0007606 biological_process sensory perception of chemical stimulus

6.19 40|14596|1619|238 GO:0032555 molecular_function purine ribonucleotide binding

6.19 40|14596|1619|238 GO:0032553 molecular_function ribonucleotide binding

6.14 0|14596|369|238 GO:0004984 molecular_function olfactory receptor activity

6.13 35|14596|1368|238 GO:0030554 molecular_function adenyl nucleotide binding

6.05 2|15182|5|239 GO:0005958 cellular_component DNA-dependent protein kinase complex

6.01 12|13581|304|218 GO:0045941 biological_process positive regulation of transcription

5.99 25|14596|882|238 GO:0005509 molecular_function calcium ion binding

5.94 3|13581|18|218 GO:0031023 biological_process microtubule organizing center organization and biogenesis

5.94 3|13581|18|218 GO:0051297 biological_process centrosome organization and biogenesis

5.94 73|15182|3582|239 GO:0044446 cellular_component intracellular organelle part

5.90 3|14596|18|238 GO:0030159 molecular_function receptor signaling complex scaffold activity

5.88 73|15182|3592|239 GO:0044422 cellular_component organelle part

5.86 12|13581|310|218 GO:0010628 biological_process positive regulation of gene expression

5.79 22|14596|753|238 GO:0016301 molecular_function kinase activity

5.79 3|13581|19|218 GO:0006473 biological_process protein amino acid acetylation

5.75 8|13581|160|218 GO:0048646 biological_process anatomical structure formation

5.73 18|14596|571|238 GO:0004672 molecular_function protein kinase activity

5.68 8|13581|162|218 GO:0000165 biological_process MAPKKK cascade

5.67 29|13581|2675|218 GO:0050896 biological_process response to stimulus

5.65 45|14596|1946|238 GO:0000166 molecular_function nucleotide binding

5.64 5|15182|874|239 GO:0005739 cellular_component mitochondrion

5.63 70|15182|5531|239 GO:0044425 cellular_component membrane part

5.60 5|14596|66|238 GO:0005262 molecular_function calcium channel activity

5.60 3|14596|20|238 GO:0032947 molecular_function protein complex scaffold

5.59 2|14596|6|238 GO:0043499 molecular_function eukaryotic cell surface binding

5.59 0|13581|341|218 GO:0007608 biological_process sensory perception of smell

5.54 40|14596|1692|238 GO:0017076 molecular_function purine nucleotide binding

5.52 12|13581|325|218 GO:0045935 biological_process "positive regulation of nucleobase, nucleoside, nucleotide and nucleic acid metabolic process"

5.50 3|13581|21|218 GO:0030261 biological_process chromosome condensation

5.50 3|13581|21|218 GO:0051016 biological_process barbed-end actin filament capping

5.50 3|13581|21|218 GO:0051693 biological_process actin filament capping

5.47 15|13581|459|218 GO:0022402 biological_process cell cycle process

5.44 5|15182|71|239 GO:0031252 cellular_component leading edge

5.44 11|14596|282|238 GO:0016563 molecular_function transcription activator activity

5.39 8|14596|168|238 GO:0003713 molecular_function transcription coactivator activity

5.37 9|13581|209|218 GO:0000087 biological_process M phase of mitotic cell cycle

5.37 7|14596|133|238 GO:0005516 molecular_function calmodulin binding

5.37 3|13581|22|218 GO:0030835 biological_process negative regulation of actin filament depolymerization

5.35 9|13581|210|218 GO:0007017 biological_process microtubule-based process

5.31 9|14596|208|238 GO:0005096 molecular_function GTPase activator activity

5.30 4|13581|737|218 GO:0006955 biological_process immune response

5.30 2|13581|7|218 GO:0006474 biological_process N-terminal protein amino acid acetylation

5.26 12|13581|337|218 GO:0022403 biological_process cell cycle phase

5.25 3|13581|23|218 GO:0030042 biological_process actin filament depolymerization

5.25 3|13581|23|218 GO:0030834 biological_process regulation of actin filament depolymerization

5.24 4|14596|45|238 GO:0008170 molecular_function N-methyltransferase activity

5.21 3|14596|23|238 GO:0003950 molecular_function NAD+ ADP-ribosyltransferase activity

5.19 16|13581|1674|218 GO:0007166 biological_process cell surface receptor linked signal transduction

5.14 139|15182|7811|239 GO:0043229 cellular_component intracellular organelle

5.14 6|15182|109|239 GO:0005875 cellular_component microtubule associated complex

5.13 139|15182|7814|239 GO:0043226 cellular_component organelle

5.11 12|15182|351|239 GO:0042995 cellular_component cell projection

5.10 7|13581|143|218 GO:0015674 biological_process "di-, tri-valent inorganic cation transport"

5.09 3|14596|24|238 GO:0008144 molecular_function drug binding

5.09 3|14596|24|238 GO:0050681 molecular_function androgen receptor binding

5.08 12|13581|346|218 GO:0010557 biological_process positive regulation of macromolecule biosynthetic process

5.07 3|15182|25|239 GO:0030863 cellular_component cortical cytoskeleton

5.03 2|13581|8|218 GO:0030069 biological_process lysogeny

5.03 2|13581|8|218 GO:0019047 biological_process provirus integration

5.02 12|13581|349|218 GO:0000278 biological_process mitotic cell cycle

4.96 13|14596|391|238 GO:0008134 molecular_function transcription factor binding

4.91 3|13581|26|218 GO:0043543 biological_process protein amino acid acylation

4.88 13|13581|402|218 GO:0010604 biological_process positive regulation of macromolecule metabolic process

4.85 2|15182|525|239 GO:0044429 cellular_component mitochondrial part

4.83 2|15182|9|239 GO:0000930 cellular_component gamma-tubulin complex

4.83 2|15182|9|239 GO:0044441 cellular_component cilium part

4.83 19|14596|682|238 GO:0016773 molecular_function "phosphotransferase activity, alcohol group as acceptor"

4.80 2|13581|9|218 GO:0006266 biological_process DNA ligation

4.80 2|13581|9|218 GO:0006303 biological_process double-strand break repair via nonhomologous end joining

4.76 3|15182|28|239 GO:0044450 cellular_component microtubule organizing center part

4.72 10|13581|277|218 GO:0000279 biological_process M phase

4.71 12|13581|366|218 GO:0009891 biological_process positive regulation of biosynthetic process

4.62 3|13581|29|218 GO:0000070 biological_process mitotic sister chromatid segregation

4.59 2|13581|10|218 GO:0018205 biological_process peptidyl-lysine modification

4.59 20|14596|752|238 GO:0030234 molecular_function enzyme regulator activity

4.58 51|13581|2480|218 GO:0006810 biological_process transport

4.57 22|13581|870|218 GO:0009653 biological_process anatomical structure morphogenesis

4.54 14|13581|470|218 GO:0006259 biological_process DNA metabolic process

4.53 3|13581|30|218 GO:0000819 biological_process sister chromatid segregation

4.51 9|14596|240|238 GO:0005083 molecular_function small GTPase regulator activity

4.44 5|14596|89|238 GO:0008022 molecular_function protein C-terminus binding

4.41 2|13581|11|218 GO:0000726 biological_process non-recombinational repair

4.41 2|13581|11|218 GO:0018409 biological_process peptide or protein amino-terminal blocking

4.40 13|13581|432|218 GO:0031325 biological_process positive regulation of cellular metabolic process

4.39 8|13581|207|218 GO:0007067 biological_process mitosis

4.37 5|13581|92|218 GO:0015980 biological_process energy derivation by oxidation of organic compounds

4.31 51|13581|2530|218 GO:0051234 biological_process establishment of localization

4.30 4|13581|61|218 GO:0000187 biological_process activation of MAPK activity

4.30 75|14596|3889|238 GO:0046872 molecular_function metal ion binding

4.27 2|15182|12|239 GO:0000123 cellular_component histone acetyltransferase complex

4.27 13|13581|441|218 GO:0009893 biological_process positive regulation of metabolic process

4.25 4|14596|61|238 GO:0035257 molecular_function nuclear hormone receptor binding

4.24 3|14596|33|238 GO:0042054 molecular_function histone methyltransferase activity

4.22 76|14596|3968|238 GO:0043167 molecular_function ion binding

4.21 2|14596|12|238 GO:0043498 molecular_function cell surface binding

4.21 1|15182|375|239 GO:0005740 cellular_component mitochondrial envelope

4.21 3|13581|34|218 GO:0030521 biological_process androgen receptor signaling pathway

4.21 3|13581|34|218 GO:0000910 biological_process cytokinesis

4.20 4|14596|62|238 GO:0051427 molecular_function hormone receptor binding

4.18 3|15182|35|239 GO:0005581 cellular_component collagen

4.17 12|13581|400|218 GO:0007243 biological_process protein kinase cascade

4.15 1|15182|1|239 GO:0005600 cellular_component collagen type XIII

4.15 1|15182|1|239 GO:0005582 cellular_component collagen type XV

4.15 1|15182|1|239 GO:0032279 cellular_component asymmetric synapse

4.15 1|15182|1|239 GO:0000799 cellular_component nuclear condensin complex

4.15 1|15182|1|239 GO:0043198 cellular_component dendritic shaft

4.15 1|15182|1|239 GO:0030936 cellular_component transmembrane collagen

4.15 1|15182|1|239 GO:0005969 cellular_component serine-pyruvate aminotransferase complex

4.13 1|13581|1|218 GO:0030423 biological_process "RNA interference, targeting of mRNA for destruction"

4.13 1|13581|1|218 GO:0030422 biological_process "RNA interference, production of siRNA"

4.13 1|13581|1|218 GO:0048245 biological_process eosinophil chemotaxis

4.13 1|13581|1|218 GO:0048243 biological_process norepinephrine secretion

4.13 1|13581|1|218 GO:0045014 biological_process negative regulation of transcription by glucose

4.13 1|13581|1|218 GO:0045013 biological_process negative regulation of transcription by carbon catabolites

4.13 1|13581|1|218 GO:0014061 biological_process regulation of norepinephrine secretion

4.13 1|13581|1|218 GO:0033693 biological_process neurofilament bundle assembly

4.13 1|13581|1|218 GO:0001988 biological_process positive regulation of heart rate in baroreceptor response to decreased systemic arterial blood pressure

4.13 1|13581|1|218 GO:0048793 biological_process pronephros development

4.13 1|13581|1|218 GO:0035247 biological_process peptidyl-arginine omega-N-methylation

4.13 1|13581|1|218 GO:0035066 biological_process positive regulation of histone acetylation

4.13 1|13581|1|218 GO:0006438 biological_process valyl-tRNA aminoacylation

4.13 1|13581|1|218 GO:0010457 biological_process centriole-centriole cohesion

4.13 1|13581|1|218 GO:0043374 biological_process "CD8-positive, alpha-beta T cell differentiation"

4.13 1|13581|1|218 GO:0030997 biological_process regulation of centriole-centriole cohesion

4.13 1|13581|1|218 GO:0030967 biological_process ER-nuclear sterol response pathway

4.13 1|13581|1|218 GO:0060112 biological_process generation of ovulation cycle rhythm

4.13 1|13581|1|218 GO:0045714 biological_process regulation of low-density lipoprotein receptor biosynthetic process

4.13 1|13581|1|218 GO:0045716 biological_process positive regulation of low-density lipoprotein receptor biosynthetic process

4.13 1|13581|1|218 GO:0030916 biological_process otic vesicle formation

4.13 1|13581|1|218 GO:0048149 biological_process behavioral response to ethanol

4.13 1|13581|1|218 GO:0016189 biological_process synaptic vesicle to endosome fusion

4.13 1|13581|1|218 GO:0019919 biological_process "peptidyl-arginine methylation, to asymmetrical-dimethyl arginine"

4.13 1|13581|1|218 GO:0006994 biological_process positive regulation of sterol regulatory element binding protein target gene transcription involved in sterol depletion response

4.12 2|15182|13|239 GO:0005865 cellular_component striated muscle thin filament

4.12 1|14596|1|238 GO:0048244 molecular_function phytanoyl-CoA dioxygenase activity

4.12 1|14596|1|238 GO:0000250 molecular_function lanosterol synthase activity

4.12 1|14596|1|238 GO:0051011 molecular_function microtubule minus-end binding

4.12 1|14596|1|238 GO:0030161 molecular_function calpain inhibitor activity

4.12 1|14596|1|238 GO:0004581 molecular_function dolichyl-phosphate beta-glucosyltransferase activity

4.12 1|14596|1|238 GO:0008892 molecular_function guanine deaminase activity

4.12 1|14596|1|238 GO:0051577 molecular_function MyoD binding

4.12 1|14596|1|238 GO:0008179 molecular_function adenylate cyclase binding

4.12 1|14596|1|238 GO:0017081 molecular_function chloride channel regulator activity

4.12 1|14596|1|238 GO:0043426 molecular_function MRF binding

4.12 1|14596|1|238 GO:0050265 molecular_function RNA uridylyltransferase activity

4.12 1|14596|1|238 GO:0031559 molecular_function oxidosqualene cyclase activity

4.12 1|14596|1|238 GO:0003721 molecular_function telomeric template RNA reverse transcriptase activity

4.12 1|14596|1|238 GO:0004832 molecular_function valine-tRNA ligase activity

4.10 6|14596|135|238 GO:0005543 molecular_function phospholipid binding

4.09 2|13581|13|218 GO:0007098 biological_process centrosome cycle

4.09 2|13581|13|218 GO:0030510 biological_process regulation of BMP signaling pathway

4.08 8|13581|936|218 GO:0002376 biological_process immune system process

4.08 5|13581|100|218 GO:0033043 biological_process regulation of organelle organization and biogenesis

4.06 3|13581|36|218 GO:0043414 biological_process biopolymer methylation

4.05 69|14596|3596|238 GO:0043169 molecular_function cation binding

4.05 1|15182|363|239 GO:0031988 cellular_component membrane-bounded vesicle

4.04 23|13581|981|218 GO:0065008 biological_process regulation of biological quality

4.04 3|15182|548|239 GO:0031975 cellular_component envelope

4.04 3|15182|548|239 GO:0031967 cellular_component organelle envelope

4.02 6|13581|777|218 GO:0009056 biological_process catabolic process

4.02 1|15182|361|239 GO:0031966 cellular_component mitochondrial membrane

4.00 54|13581|2772|218 GO:0044260 biological_process cellular macromolecule metabolic process

3.99 3|13581|37|218 GO:0051261 biological_process protein depolymerization

3.98 1|15182|358|239 GO:0016023 cellular_component cytoplasmic membrane-bounded vesicle

3.98 3|15182|38|239 GO:0030425 cellular_component dendrite

3.97 50|13581|3743|218 GO:0009058 biological_process biosynthetic process

3.96 8|13581|227|218 GO:0051301 biological_process cell division

3.95 2|13581|14|218 GO:0001937 biological_process negative regulation of endothelial cell proliferation

3.95 2|13581|14|218 GO:0031365 biological_process N-terminal protein amino acid modification

3.93 3|13581|38|218 GO:0007015 biological_process actin filament organization

3.92 4|14596|68|238 GO:0005200 molecular_function structural constituent of cytoskeleton

3.90 11|13581|370|218 GO:0009887 biological_process organ morphogenesis

3.88 5|14596|104|238 GO:0003714 molecular_function transcription corepressor activity

3.88 19|15182|799|239 GO:0031981 cellular_component nuclear lumen

3.87 5|13581|682|218 GO:0006508 biological_process proteolysis

3.86 3|13581|39|218 GO:0035107 biological_process appendage morphogenesis

3.86 3|13581|39|218 GO:0035108 biological_process limb morphogenesis

3.86 3|13581|39|218 GO:0048736 biological_process appendage development

3.86 3|13581|39|218 GO:0060173 biological_process limb development

3.82 49|13581|2516|218 GO:0044267 biological_process cellular protein metabolic process

3.80 3|13581|40|218 GO:0005977 biological_process glycogen metabolic process

3.79 4|13581|72|218 GO:0043406 biological_process positive regulation of MAP kinase activity

3.76 20|13581|852|218 GO:0051641 biological_process cellular localization

3.74 3|13581|41|218 GO:0006073 biological_process glucan metabolic process

3.74 41|14596|3059|238 GO:0003676 molecular_function nucleic acid binding

3.74 11|15182|389|239 GO:0044451 cellular_component nucleoplasm part

3.74 9|13581|285|218 GO:0051726 biological_process regulation of cell cycle

3.74 99|15182|6898|239 GO:0043227 cellular_component membrane-bounded organelle

3.73 99|15182|6896|239 GO:0043231 cellular_component intracellular membrane-bounded organelle

3.71 2|13581|16|218 GO:0007589 biological_process body fluid secretion

3.70 3|14596|41|238 GO:0008276 molecular_function protein methyltransferase activity

3.68 3|13581|42|218 GO:0008016 biological_process regulation of heart contraction

3.68 43|13581|3216|218 GO:0044249 biological_process cellular biosynthetic process

3.68 87|13581|4866|218 GO:0043283 biological_process biopolymer metabolic process

3.68 2|14596|16|238 GO:0016455 molecular_function RNA polymerase II transcription mediator activity

3.66 17|13581|701|218 GO:0015031 biological_process protein transport

3.65 17|13581|702|218 GO:0045184 biological_process establishment of protein localization

3.65 24|14596|1947|238 GO:0016787 molecular_function hydrolase activity

3.63 4|14596|75|238 GO:0003777 molecular_function microtubule motor activity

3.63 54|13581|2865|218 GO:0051179 biological_process localization

3.63 3|13581|43|218 GO:0032259 biological_process methylation

3.63 49|13581|2564|218 GO:0019538 biological_process protein metabolic process

3.62 19|13581|815|218 GO:0051649 biological_process establishment of localization in cell

3.62 19|13581|815|218 GO:0033036 biological_process macromolecule localization

3.62 4|15182|78|239 GO:0000793 cellular_component condensed chromosome

3.60 5|13581|115|218 GO:0006323 biological_process DNA packaging

3.60 2|13581|17|218 GO:0001756 biological_process somitogenesis

3.60 2|13581|17|218 GO:0009612 biological_process response to mechanical stimulus

3.59 4|14596|76|238 GO:0035091 molecular_function phosphoinositide binding

3.59 3|14596|43|238 GO:0016763 molecular_function "transferase activity, transferring pentosyl groups"

3.58 18|13581|765|218 GO:0008104 biological_process protein localization

3.57 3|13581|44|218 GO:0040029 biological_process "regulation of gene expression, epigenetic"

3.56 7|14596|199|238 GO:0042803 molecular_function protein homodimerization activity

3.55 8|13581|249|218 GO:0006091 biological_process generation of precursor metabolites and energy

3.54 24|15182|1128|239 GO:0044428 cellular_component nuclear part

3.54 8|14596|246|238 GO:0016757 molecular_function "transferase activity, transferring glycosyl groups"

3.53 29|13581|1397|218 GO:0007242 biological_process intracellular signaling cascade

3.51 11|15182|407|239 GO:0030054 cellular_component cell junction

3.49 5|13581|119|218 GO:0048598 biological_process embryonic morphogenesis

3.47 1|15182|2|239 GO:0032391 cellular_component photoreceptor connecting cilium

3.47 1|15182|2|239 GO:0031213 cellular_component RSF complex

3.47 1|15182|2|239 GO:0042641 cellular_component actomyosin

3.47 1|15182|2|239 GO:0045179 cellular_component apical cortex

3.47 1|15182|2|239 GO:0032807 cellular_component DNA ligase IV complex

3.47 8|14596|250|238 GO:0005261 molecular_function cation channel activity

3.47 3|13581|46|218 GO:0006112 biological_process energy reserve metabolic process

3.47 3|13581|46|218 GO:0008064 biological_process regulation of actin polymerization and/or depolymerization

3.47 2|14596|18|238 GO:0004709 molecular_function MAP kinase kinase kinase activity

3.46 4|13581|81|218 GO:0051028 biological_process mRNA transport

3.46 115|13581|6687|218 GO:0065007 biological_process biological regulation

3.45 1|13581|2|218 GO:0031050 biological_process dsRNA fragmentation

3.45 1|13581|2|218 GO:0031058 biological_process positive regulation of histone modification

3.45 1|13581|2|218 GO:0048570 biological_process notochord morphogenesis

3.45 1|13581|2|218 GO:0032225 biological_process "regulation of synaptic transmission, dopaminergic"

3.45 1|13581|2|218 GO:0035104 biological_process positive regulation of sterol regulatory element binding protein target gene transcription

3.45 1|13581|2|218 GO:0035196 biological_process "gene silencing by miRNA, production of miRNAs"

3.45 1|13581|2|218 GO:0035195 biological_process gene silencing by miRNA

3.45 1|13581|2|218 GO:0001982 biological_process baroreceptor response to decreased systemic arterial blood pressure

3.45 1|13581|2|218 GO:0035246 biological_process peptidyl-arginine N-methylation

3.45 1|13581|2|218 GO:0010216 biological_process maintenance of DNA methylation

3.45 1|13581|2|218 GO:0015939 biological_process pantothenate metabolic process

3.45 1|13581|2|218 GO:0007231 biological_process osmosensory signaling pathway

3.45 1|13581|2|218 GO:0035065 biological_process regulation of histone acetylation

3.45 1|13581|2|218 GO:0045722 biological_process positive regulation of gluconeogenesis

3.45 1|13581|2|218 GO:0050434 biological_process positive regulation of viral transcription

3.45 1|13581|2|218 GO:0045939 biological_process negative regulation of steroid metabolic process

3.45 1|13581|2|218 GO:0017158 biological_process regulation of calcium ion-dependent exocytosis

3.45 1|13581|2|218 GO:0045541 biological_process negative regulation of cholesterol biosynthetic process

3.45 1|13581|2|218 GO:0008090 biological_process retrograde axon cargo transport

3.45 1|13581|2|218 GO:0006608 biological_process snRNP protein import into nucleus

3.45 1|13581|2|218 GO:0045713 biological_process low-density lipoprotein receptor biosynthetic process

3.45 1|13581|2|218 GO:0032898 biological_process neurotrophin production

3.45 1|13581|2|218 GO:0032933 biological_process SREBP-mediated signaling pathway

3.45 1|13581|2|218 GO:0032902 biological_process nerve growth factor production

3.45 1|13581|2|218 GO:0006991 biological_process response to sterol depletion

3.45 1|13581|2|218 GO:0045110 biological_process intermediate filament bundle assembly

3.45 1|13581|2|218 GO:0019896 biological_process axon transport of mitochondrion

3.45 111|13581|7387|218 GO:0008152 biological_process metabolic process

3.44 1|14596|2|238 GO:0004336 molecular_function galactosylceramidase activity

3.44 1|14596|2|238 GO:0047223 molecular_function "beta-1,3-galactosyl-O-glycosyl-glycoprotein beta-1,3-N-acetylglucosaminyltransferase activity"

3.44 1|14596|2|238 GO:0004557 molecular_function alpha-galactosidase activity

3.44 1|14596|2|238 GO:0005536 molecular_function glucose binding

3.44 1|14596|2|238 GO:0004473 molecular_function malate dehydrogenase (oxaloacetate-decarboxylating) (NADP+) activity

3.44 1|14596|2|238 GO:0042975 molecular_function peroxisome proliferator activated receptor binding

3.44 1|14596|2|238 GO:0035241 molecular_function protein-arginine omega-N monomethyltransferase activity

3.44 1|14596|2|238 GO:0004098 molecular_function cerebroside-sulfatase activity

3.44 1|14596|2|238 GO:0002054 molecular_function nucleobase binding

3.44 1|14596|2|238 GO:0002060 molecular_function purine binding

3.44 1|14596|2|238 GO:0017034 molecular_function Rap guanyl-nucleotide exchange factor activity

3.44 1|14596|2|238 GO:0048155 molecular_function S100 alpha binding

3.44 1|14596|2|238 GO:0003827 molecular_function "alpha-1,3-mannosylglycoprotein 2-beta-N-acetylglucosaminyltransferase activity"

3.43 10|14596|350|238 GO:0005216 molecular_function ion channel activity

3.42 4|13581|82|218 GO:0006606 biological_process protein import into nucleus

3.42 3|13581|47|218 GO:0051494 biological_process negative regulation of cytoskeleton organization and biogenesis

3.41 42|14596|2163|238 GO:0008270 molecular_function zinc ion binding

3.41 9|14596|302|238 GO:0046873 molecular_function metal ion transmembrane transporter activity

3.38 3|14596|47|238 GO:0034061 molecular_function DNA polymerase activity

3.37 3|13581|48|218 GO:0030832 biological_process regulation of actin filament length

3.36 4|13581|84|218 GO:0051170 biological_process nuclear import

3.34 2|15182|20|239 GO:0016528 cellular_component sarcoplasm

3.34 2|15182|20|239 GO:0016529 cellular_component sarcoplasmic reticulum

3.34 5|14596|123|238 GO:0019842 molecular_function vitamin binding

3.33 3|14596|48|238 GO:0008017 molecular_function microtubule binding

3.32 4|13581|85|218 GO:0006817 biological_process phosphate transport

3.32 3|13581|49|218 GO:0006338 biological_process chromatin remodeling

3.32 17|13581|743|218 GO:0006811 biological_process ion transport

3.32 3|15182|50|239 GO:0044463 cellular_component cell projection part

3.32 188|13581|11298|218 GO:0009987 biological_process cellular process

3.31 10|14596|359|238 GO:0022838 molecular_function substrate specific channel activity

3.31 4|15182|87|239 GO:0043005 cellular_component neuron projection

3.31 96|15182|6514|239 GO:0016020 cellular_component membrane

3.31 2|13581|20|218 GO:0008213 biological_process protein amino acid alkylation

3.31 2|13581|20|218 GO:0006479 biological_process protein amino acid methylation

3.28 2|14596|20|238 GO:0005328 molecular_function neurotransmitter:sodium symporter activity

3.28 2|14596|20|238 GO:0003964 molecular_function RNA-directed DNA polymerase activity

3.28 3|13581|50|218 GO:0030518 biological_process steroid hormone receptor signaling pathway

3.27 13|14596|520|238 GO:0008324 molecular_function cation transmembrane transporter activity

3.24 3|14596|50|238 GO:0004702 molecular_function receptor signaling protein serine/threonine kinase activity

3.24 66|13581|3739|218 GO:0007154 biological_process cell communication

3.23 3|13581|51|218 GO:0032535 biological_process regulation of cellular component size

3.23 4|13581|88|218 GO:0051493 biological_process regulation of cytoskeleton organization and biogenesis

3.23 2|13581|21|218 GO:0007257 biological_process activation of JNK activity

3.22 5|13581|130|218 GO:0007389 biological_process pattern specification process

3.22 7|13581|222|218 GO:0051056 biological_process regulation of small GTPase mediated signal transduction

3.21 5|15182|133|239 GO:0005667 cellular_component transcription factor complex

3.20 10|14596|369|238 GO:0022803 molecular_function passive transmembrane transporter activity

3.20 10|14596|369|238 GO:0015267 molecular_function channel activity

3.20 5|13581|131|218 GO:0001525 biological_process angiogenesis

3.17 7|13581|745|218 GO:0008283 biological_process cell proliferation

3.16 5|15182|135|239 GO:0034702 cellular_component ion channel complex

3.15 2|13581|22|218 GO:0019059 biological_process initiation of viral infection

3.15 2|13581|22|218 GO:0001936 biological_process regulation of endothelial cell proliferation

3.15 2|13581|22|218 GO:0015074 biological_process DNA integration

3.14 4|13581|91|218 GO:0051236 biological_process establishment of RNA localization

3.14 4|13581|91|218 GO:0050658 biological_process RNA transport

3.14 4|13581|91|218 GO:0050657 biological_process nucleic acid transport

3.13 5|13581|134|218 GO:0007605 biological_process sensory perception of sound

3.13 5|13581|134|218 GO:0050954 biological_process sensory perception of mechanical stimulus

3.12 96|13581|5682|218 GO:0043170 biological_process macromolecule metabolic process

3.12 2|14596|22|238 GO:0042287 molecular_function MHC protein binding

3.11 4|13581|92|218 GO:0000226 biological_process microtubule cytoskeleton organization and biogenesis

3.11 4|13581|92|218 GO:0006403 biological_process RNA localization

3.11 3|14596|53|238 GO:0030170 molecular_function pyridoxal phosphate binding

3.11 5|13581|135|218 GO:0045860 biological_process positive regulation of protein kinase activity

3.11 3|13581|54|218 GO:0010639 biological_process negative regulation of organelle organization and biogenesis

3.10 2|15182|23|239 GO:0000781 cellular_component "chromosome, telomeric region"

3.10 2|15182|23|239 GO:0000922 cellular_component spindle pole

3.09 21|13581|1011|218 GO:0048522 biological_process positive regulation of cellular process

3.09 6|14596|654|238 GO:0016491 molecular_function oxidoreductase activity

3.08 1|15182|3|239 GO:0001891 cellular_component phagocytic cup

3.08 1|15182|3|239 GO:0033596 cellular_component TSC1-TSC2 complex

3.08 1|15182|3|239 GO:0035253 cellular_component ciliary rootlet

3.08 1|15182|3|239 GO:0008537 cellular_component proteasome activator complex

3.07 1|15182|288|239 GO:0019866 cellular_component organelle inner membrane

3.07 3|13581|55|218 GO:0001666 biological_process response to hypoxia

3.07 3|13581|55|218 GO:0003015 biological_process heart process

3.07 3|13581|55|218 GO:0060047 biological_process heart contraction

3.07 1|13581|3|218 GO:0046015 biological_process regulation of transcription by glucose

3.07 1|13581|3|218 GO:0031056 biological_process regulation of histone modification

3.07 1|13581|3|218 GO:0030157 biological_process pancreatic juice secretion

3.07 1|13581|3|218 GO:0018216 biological_process peptidyl-arginine methylation

3.07 1|13581|3|218 GO:0046321 biological_process positive regulation of fatty acid oxidation

3.07 1|13581|3|218 GO:0001958 biological_process endochondral ossification

3.07 1|13581|3|218 GO:0045990 biological_process regulation of transcription by carbon catabolites

3.07 1|13581|3|218 GO:0010460 biological_process positive regulation of heart rate

3.07 1|13581|3|218 GO:0045663 biological_process positive regulation of myoblast differentiation

3.07 1|13581|3|218 GO:0050901 biological_process leukocyte tethering or rolling

3.07 1|13581|3|218 GO:0016246 biological_process RNA interference

3.07 1|13581|3|218 GO:0031573 biological_process intra-S DNA damage checkpoint

3.07 1|13581|3|218 GO:0032692 biological_process negative regulation of interleukin-1 production

3.07 1|13581|3|218 GO:0032691 biological_process negative regulation of interleukin-1 beta production

3.07 1|13581|3|218 GO:0032651 biological_process regulation of interleukin-1 beta production

3.07 1|13581|3|218 GO:0032720 biological_process negative regulation of tumor necrosis factor production

3.07 1|13581|3|218 GO:0046782 biological_process regulation of viral transcription

3.06 4|13581|94|218 GO:0007018 biological_process microtubule-based movement

3.05 1|14596|3|238 GO:0004348 molecular_function glucosylceramidase activity

3.05 1|14596|3|238 GO:0004525 molecular_function ribonuclease III activity

3.05 1|14596|3|238 GO:0008384 molecular_function IkappaB kinase activity

3.05 1|14596|3|238 GO:0017159 molecular_function pantetheine hydrolase activity

3.05 1|14596|3|238 GO:0017112 molecular_function Rab guanyl-nucleotide exchange factor activity

3.05 1|14596|3|238 GO:0046582 molecular_function Rap GTPase activator activity

3.05 1|14596|3|238 GO:0004862 molecular_function cAMP-dependent protein kinase inhibitor activity

3.05 2|14596|23|238 GO:0005326 molecular_function neurotransmitter transporter activity

3.05 60|13581|3456|218 GO:0006139 biological_process "nucleobase, nucleoside, nucleotide and nucleic acid metabolic process"

3.04 5|13581|138|218 GO:0033674 biological_process positive regulation of kinase activity

3.04 5|14596|136|238 GO:0003774 molecular_function motor activity

3.03 3|13581|56|218 GO:0019058 biological_process viral infectious cycle

3.03 1|13581|279|218 GO:0009607 biological_process response to biotic stimulus

3.01 7|13581|236|218 GO:0009790 biological_process embryonic development

3.00 1|14596|273|238 GO:0005506 molecular_function iron ion binding

3.00 4|15182|98|239 GO:0045177 cellular_component apical part of cell

3.00 6|13581|652|218 GO:0044248 biological_process cellular catabolic process

3.00 2|13581|24|218 GO:0043507 biological_process positive regulation of JNK activity

3.00 34|13581|1831|218 GO:0048856 biological_process anatomical structure development

2.99 3|13581|57|218 GO:0009952 biological_process anterior/posterior pattern formation

2.99 3|13581|57|218 GO:0030522 biological_process intracellular receptor-mediated signaling pathway

2.99 3|13581|57|218 GO:0043624 biological_process cellular protein complex disassembly

2.98 5|13581|141|218 GO:0051347 biological_process positive regulation of transferase activity

2.98 10|14596|905|238 GO:0003700 molecular_function transcription factor activity

2.98 2|14596|24|238 GO:0030374 molecular_function ligand-dependent nuclear receptor transcription coactivator activity

2.97 107|13581|6821|218 GO:0044237 biological_process cellular metabolic process

2.96 40|13581|2773|218 GO:0019222 biological_process regulation of metabolic process

2.95 3|13581|58|218 GO:0034623 biological_process cellular macromolecular complex disassembly

2.95 3|13581|58|218 GO:0043241 biological_process protein complex disassembly

2.95 3|13581|58|218 GO:0010564 biological_process regulation of cell cycle process

2.95 3|13581|58|218 GO:0032956 biological_process regulation of actin cytoskeleton organization and biogenesis

2.94 14|13581|626|218 GO:0065009 biological_process regulation of molecular function

2.94 104|13581|6364|218 GO:0050789 biological_process regulation of biological process

2.92 22|15182|1130|239 GO:0043233 cellular_component organelle lumen

2.92 22|15182|1130|239 GO:0031974 cellular_component membrane-enclosed lumen

2.92 3|15182|60|239 GO:0048475 cellular_component coated membrane

2.92 3|15182|60|239 GO:0030117 cellular_component membrane coat

2.92 47|13581|3172|218 GO:0009059 biological_process macromolecule biosynthetic process

2.91 106|13581|6634|218 GO:0044238 biological_process primary metabolic process

2.91 99|13581|6192|218 GO:0050794 biological_process regulation of cellular process

2.91 78|14596|4704|238 GO:0003824 molecular_function catalytic activity

2.91 31|14596|2174|238 GO:0003677 molecular_function DNA binding

2.90 46|14596|2604|238 GO:0046914 molecular_function transition metal ion binding

2.90 1|13581|269|218 GO:0022008 biological_process neurogenesis

2.89 48|13581|3215|218 GO:0010467 biological_process gene expression

2.89 8|14596|292|238 GO:0016887 molecular_function ATPase activity

2.89 5|13581|146|218 GO:0045786 biological_process negative regulation of cell cycle

2.88 3|13581|60|218 GO:0008154 biological_process actin polymerization and/or depolymerization

2.87 2|13581|26|218 GO:0006090 biological_process pyruvate metabolic process

2.87 2|13581|26|218 GO:0001935 biological_process endothelial cell proliferation

2.87 2|13581|26|218 GO:0033500 biological_process carbohydrate homeostasis

2.87 2|13581|26|218 GO:0035282 biological_process segmentation

2.87 2|13581|26|218 GO:0042593 biological_process glucose homeostasis

2.87 2|13581|26|218 GO:0043506 biological_process regulation of JNK activity

2.87 36|13581|2491|218 GO:0009889 biological_process regulation of biosynthetic process

2.87 70|15182|4409|239 GO:0005634 cellular_component nucleus

2.86 15|14596|687|238 GO:0015075 molecular_function ion transmembrane transporter activity

2.86 4|13581|102|218 GO:0043405 biological_process regulation of MAP kinase activity

2.85 3|13581|61|218 GO:0007059 biological_process chromosome segregation

2.85 3|13581|61|218 GO:0032970 biological_process regulation of actin filament-based process

2.85 3|13581|61|218 GO:0032984 biological_process macromolecular complex disassembly

2.84 2|14596|26|238 GO:0018024 molecular_function histone-lysine N-methyltransferase activity

2.84 2|14596|26|238 GO:0004468 molecular_function lysine N-acetyltransferase activity

2.84 2|14596|26|238 GO:0004402 molecular_function histone acetyltransferase activity

2.84 2|14596|26|238 GO:0016278 molecular_function lysine N-methyltransferase activity

2.84 2|14596|26|238 GO:0016279 molecular_function protein-lysine N-methyltransferase activity

2.84 14|15182|654|239 GO:0005654 cellular_component nucleoplasm

2.84 2|15182|27|239 GO:0030131 cellular_component clathrin adaptor complex

2.84 57|13581|3417|218 GO:0007165 biological_process signal transduction

2.83 39|13581|2649|218 GO:0060255 biological_process regulation of macromolecule metabolic process

2.83 7|13581|249|218 GO:0006281 biological_process DNA repair

2.83 19|15182|1483|239 GO:0031090 cellular_component organelle membrane

2.83 10|14596|406|238 GO:0000287 molecular_function magnesium ion binding

2.83 40|13581|2703|218 GO:0031323 biological_process regulation of cellular metabolic process

2.82 1|15182|268|239 GO:0005743 cellular_component mitochondrial inner membrane

2.82 36|13581|2463|218 GO:0010556 biological_process regulation of macromolecule biosynthetic process

2.81 1|15182|4|239 GO:0005862 cellular_component muscle thin filament tropomyosin

2.81 1|15182|4|239 GO:0000796 cellular_component condensin complex

2.81 1|15182|4|239 GO:0008274 cellular_component gamma-tubulin ring complex

2.81 1|15182|4|239 GO:0000931 cellular_component gamma-tubulin large complex

2.81 1|15182|4|239 GO:0035085 cellular_component cilium axoneme

2.81 59|15182|3780|239 GO:0044444 cellular_component cytoplasmic part

2.81 2|13581|27|218 GO:0050804 biological_process regulation of synaptic transmission

2.81 4|13581|104|218 GO:0015931 biological_process "nucleobase, nucleoside, nucleotide and nucleic acid transport"

2.79 5|13581|151|218 GO:0015698 biological_process inorganic anion transport

2.79 1|13581|4|218 GO:0032611 biological_process interleukin-1 beta production

2.79 1|13581|4|218 GO:0051149 biological_process positive regulation of muscle cell differentiation

2.79 1|13581|4|218 GO:0032455 biological_process nerve growth factor processing

2.79 1|13581|4|218 GO:0001678 biological_process cellular glucose homeostasis

2.79 1|13581|4|218 GO:0019083 biological_process viral transcription

2.79 1|13581|4|218 GO:0019080 biological_process viral genome expression

2.79 1|13581|4|218 GO:0006111 biological_process regulation of gluconeogenesis

2.79 1|13581|4|218 GO:0035194 biological_process posttranscriptional gene silencing by RNA

2.79 1|13581|4|218 GO:0001978 biological_process regulation of systemic arterial blood pressure by carotid sinus baroreceptor feedback

2.79 1|13581|4|218 GO:0007500 biological_process mesodermal cell fate determination

2.79 1|13581|4|218 GO:0003025 biological_process regulation of systemic arterial blood pressure by baroreceptor feedback

2.79 1|13581|4|218 GO:0006349 biological_process genetic imprinting

2.79 1|13581|4|218 GO:0007253 biological_process cytoplasmic sequestering of NF-kappaB

2.79 1|13581|4|218 GO:0046885 biological_process regulation of hormone biosynthetic process

2.79 1|13581|4|218 GO:0060017 biological_process parathyroid gland development

2.79 1|13581|4|218 GO:0030903 biological_process notochord development

2.79 1|13581|4|218 GO:0048096 biological_process chromatin-mediated maintenance of transcription

2.79 1|13581|4|218 GO:0032652 biological_process regulation of interleukin-1 production

2.79 1|13581|4|218 GO:0032715 biological_process negative regulation of interleukin-6 production

2.79 1|13581|4|218 GO:0042416 biological_process dopamine biosynthetic process

2.79 1|13581|4|218 GO:0032799 biological_process low-density lipoprotein receptor metabolic process

2.79 1|13581|4|218 GO:0016441 biological_process posttranscriptional gene silencing

2.79 45|13581|2962|218 GO:0043284 biological_process biopolymer biosynthetic process

2.79 8|13581|306|218 GO:0006974 biological_process response to DNA damage stimulus

2.79 35|13581|2389|218 GO:0019219 biological_process "regulation of nucleobase, nucleoside, nucleotide and nucleic acid metabolic process"

2.79 4|13581|105|218 GO:0017038 biological_process protein import

2.78 1|13581|260|218 GO:0030163 biological_process protein catabolic process

2.78 2|15182|28|239 GO:0030119 cellular_component AP-type membrane coat adaptor complex

2.78 52|13581|3333|218 GO:0032501 biological_process multicellular organismal process

2.78 3|13581|63|218 GO:0022415 biological_process viral reproductive process

2.78 1|14596|4|238 GO:0005522 molecular_function profilin binding

2.78 1|14596|4|238 GO:0015643 molecular_function toxin binding

2.78 1|14596|4|238 GO:0004470 molecular_function malic enzyme activity

2.78 1|14596|4|238 GO:0005432 molecular_function calcium:sodium antiporter activity

2.78 1|14596|4|238 GO:0032052 molecular_function bile acid binding

2.78 1|14596|4|238 GO:0008538 molecular_function proteasome activator activity

2.78 1|14596|4|238 GO:0048154 molecular_function S100 beta binding

2.78 1|14596|4|238 GO:0004704 molecular_function NF-kappaB-inducing kinase activity

2.78 1|14596|4|238 GO:0003720 molecular_function telomerase activity

2.77 49|13581|2952|218 GO:0032502 biological_process developmental process

2.76 50|15182|3122|239 GO:0005886 cellular_component plasma membrane

2.76 37|13581|2483|218 GO:0010468 biological_process regulation of gene expression

2.75 14|15182|1148|239 GO:0031226 cellular_component intrinsic to plasma membrane

2.75 2|13581|28|218 GO:0006278 biological_process RNA-dependent DNA replication

2.75 2|13581|28|218 GO:0016458 biological_process gene silencing

2.75 6|13581|616|218 GO:0042221 biological_process response to chemical stimulus

2.75 3|13581|64|218 GO:0044264 biological_process cellular polysaccharide metabolic process

2.74 14|13581|659|218 GO:0046907 biological_process intracellular transport

2.73 11|13581|482|218 GO:0016192 biological_process vesicle-mediated transport

2.73 5|13581|546|218 GO:0006952 biological_process defense response

2.73 5|13581|155|218 GO:0048514 biological_process blood vessel morphogenesis

2.71 45|13581|2754|218 GO:0016070 biological_process RNA metabolic process

2.70 6|14596|204|238 GO:0016564 molecular_function transcription repressor activity

2.70 2|13581|29|218 GO:0032507 biological_process maintenance of protein location in cell

2.70 2|13581|29|218 GO:0051969 biological_process regulation of transmission of nerve impulse

2.70 35|13581|2326|218 GO:0045449 biological_process regulation of transcription

2.69 14|15182|1132|239 GO:0005887 cellular_component integral to plasma membrane

2.69 3|13581|66|218 GO:0007519 biological_process skeletal muscle development

2.69 3|13581|66|218 GO:0005976 biological_process polysaccharide metabolic process

2.69 3|14596|65|238 GO:0015631 molecular_function tubulin binding

2.68 21|13581|1110|218 GO:0048518 biological_process positive regulation of biological process

2.68 11|13581|489|218 GO:0006812 biological_process cation transport

2.68 1|13581|252|218 GO:0048699 biological_process generation of neurons

2.67 37|13581|2413|218 GO:0006350 biological_process transcription

2.67 2|15182|30|239 GO:0030286 cellular_component dynein complex

2.66 13|13581|613|218 GO:0006366 biological_process transcription from RNA polymerase II promoter

2.66 7|13581|264|218 GO:0046903 biological_process secretion

2.66 3|13581|67|218 GO:0007254 biological_process JNK cascade
